# Unique Solid Phase Microextraction Sampler Reveals Distinctive Biogeochemical Profiles among Various Deep-Sea Hydrothermal Vents

**DOI:** 10.1038/s41598-020-58418-4

**Published:** 2020-01-28

**Authors:** Jonathan James Grandy, Bora Onat, Verena Tunnicliffe, David A. Butterfield, Janusz Pawliszyn

**Affiliations:** 1 Department of Chemistry, University of Waterloo, 200 University Avenue West, Waterloo, ON Canada; 2 Department of Biology and School of Earth & Ocean Sciences, University of Victoria, Victoria, BC Canada; 3 NOAA/PMEL, 7600 Sand Pt Way, Seattle, Washington, NE 98115-6349 USA; 4 JISAO, University of Washington, Washington, 98115-6349 USA

**Keywords:** Biochemistry, Biological techniques, Biogeochemistry, Ocean sciences, Chemistry

## Abstract

Current methods for biochemical and biogeochemical analysis of the deep-sea hydrothermal vent ecosystems rely on water sample recovery, or *in situ* analysis using underwater instruments with limited range of analyte detection and limited sensitivity. Even in cases where large quantities of sample are recovered, labile dissolved organic compounds may not be detected due to time delays between sampling and preservation. Here, we present a novel approach for *in situ* extraction of organic compounds from hydrothermal vent fluids through a unique solid phase microextraction (SPME) sampler. These samplers were deployed to sample effluent of vents on sulphide chimneys, located on Axial Seamount in the North-East Pacific, in the Urashima field on the southern Mariana back-arc, and at the Hafa Adai site in the central Mariana back-arc. Among the compounds that were extracted, a wide range of unique organic compounds, including labile dissolved organic sulfur compounds, were detected through high-resolution LC-MS/MS, among which were biomarkers of anammox bacteria, fungi, and lower animals. This report is the first to show that SPME can contribute to a broader understanding of deep sea ecology and biogeochemical cycles in hydrothermal vent ecosystems.

## Introduction

In the last three decades, study of hydrothermal vents has been an attractive topic in ecology and environmental sciences due to the richness of biological activity in these systems and their contribution to global biogeochemical cycles. Previous studies postulate that hydrothermal vents are a significant source of labile organic compounds, indicating a fertile environment for heterotrophic microbial activity^1^. Even though a substantial amount of analysis has been carried out on the biogeochemical cycles and the metabolic profiles of organisms that live in hydrothermal vent ecosystems, previous methods have relied either on the cultivation of acquired microorganisms and macroorganisms *in vitro*, or on the analysis of recovered liquid and solid samples to understand the biogeochemical processes in these settings. Especially when the concentrations and the variety of dissolved organic carbon (DOC) is assessed, liquid sampling has been the most commonly used method. However, these methods do not always represent the biogeochemical profiles of the vents, as they are often unable to provide a true ‘snapshot’ of such environments nor capture labile DOCs that may have important functions for marine organisms.

To date, analyses of biotic or abiotic compounds in hydrothermal vents have been executed either *in situ* through analytical instruments such as Raman spectrophotometers^2^ and mass spectrometers^3^ on remotely operated vehicles (ROVs), or *ex situ*, using samples acquired directly from vent sites^1^. Although *in situ* methods enable direct analysis of analytes present in these environments, including labile compounds, the range of analytes that can be captured via such methods is still limited to metals, inorganic nutrients, dissolved gases, and volatile compounds. Further, as analytical instruments deployed onboard ROVs must be designed to resist extreme pressures and temperatures, they are difficult and expensive to build and maintain. Detailed, high-resolution analysis of non-volatile organic compounds present in deep ocean environments has only been possible to date through *ex situ* chromatographic separation and high-resolution mass spectrometry. Traditional sample preparation techniques generally used shipboard for extraction of such compounds require very large sample volumes to detect and quantify trace level DOCs.

A similar problem arises when dissolved organic sulfur (DOS) in hydrothermal vent sites is targeted for analysis. DOSs are hard to identify and quantify for two reasons: their half-life in these waters can go down to minutes^4^, and heterotrophic bacterial scavenging significantly reduces their initial concentrations^5,6^. For example, the study by Landa *et al*. on the importance of sulfur metabolites on carbon flux between phytoplankton and bacteria reported the quantification of only one DOS called dimethylsulfoniopropionate (DMSP) once both organisms were cultivated in a co-culture medium *in vitro*. The expression of organic sulfur pathways and the changes in the sulfur metabolic activity in the bacterial strain *Ruegeria pomeroyi* was only successfully analyzed through patterns of expression of genes related with the sulfur metabolite uptake by bacteria^7^. The gene expression patterns revealed that *R. pomeroyi* growth was likely supported by the organosulfur compounds 2,3-dihydroxypropane-1-sulfonate (DHPS), taurine, *N-*acetyltaurine, and dimethylsulfoniopropionate (DMSP) even though only DMSP was used to determine the concentration in the media. This limitation was primarily due to very short half-lives of these organic compounds.

Several studies by Dittmar and coworkers have focused on labile dissolved organic matter (DOM) and its extraction through solid phase extraction (SPE)^8,9^. Diffuse hydrothermal systems are good targets due to the presence of a rich DOM component which is further modified by microbial processes^9,10^. One study by this group revealed that 83% of this DOM extracted from water samples was non-labile. The labile portion was metabolized by bacteria in only a few hours and labile compounds with shorter half-lives showed higher H/C ratios and lower O/C ratios. The transport of the vent fluids and the microbial activity in these samples were reported to be the main reasons labile compounds are lost before analysis^9^. For these reasons, only about 5% of the molecular structure of DOM in marine environments is known^11^.

The shortcomings of exhaustive methods in terms of the analysis of DOMs and DOSs in marine settings makes it important to apply a method where all labile organic compounds can be extracted and annotated efficiently. The objective of this report is to reveal detailed information, through the use of *in silico* annotation, on molecular formulas and nomenclature of organic compounds that were significantly higher in hydrothermal vent fluids than background oceanic water. Briefly, in our methodological setup, solid phase microextraction (SPME) combined sampling, sample clean-up, and analyte pre-concentration in one step by means of chemical extraction, a feature that makes the technique highly advantageous for applications entailing environments where long-term sampling is not feasible, and where sample preparation is tedious. Since the chemical extraction abilities of SPME effectively enable the isolation of DOC from the sample matrix upon extraction, it allowed for the extraction and on-coating stabilization of otherwise labile DOMs and DOS on site, a feature currently not possible through classical sampling techniques^12^. Even though it does not allow an exhaustive extraction of organic compounds from matrices, SPME makes the analysis of labile organic compounds possible as these compounds are extracted *in situ* through the sorbent coating on SPME devices.

The efficiency of SPME on extracting labile metabolites is known. In an *in vivo* study of mice metabolome, SPME was demonstrated to enable the capture of metabolites (e.g. glutathione) characterized by fast turnover rates and high instability not otherwise detectable via exhaustive methods such as solvent precipitation and ultrafiltration^13^. In this regard, headspace SPME has already been successfully applied to detect volatile compounds released by volcanic-hydrothermal systems^14^. As reported by Tassi *et al*., characterization of volatile organic carbons (VOCs) released by volcanic-hydrothermal systems by SPME and solid trap (ST) methods followed by GC-MS analysis yielded very similar VOC contents^15^.

We describe the use of a novel SPME device coined as the self-sealing thin film coated bolt sampler and demonstrate our proof-of-concept through *in situ* SPME sampling of temperature fluids venting through sulfide chimneys at three hydrothermal vent sites: Axial Seamount (El Gordo Vent) in the North-East Pacific, the Urashima site (Ultranochichi Vent) on the southern Mariana back-arc spreading centre, and the Hafa Adai site (Alba Vent) in the central Mariana back-arc. On-site extractions were carried out with novel and easy-to-use TF-SPME coated bolt samplers with sorbent coatings specifically designed for labile DOC and broad-spectrum metabolomics applications. The large number of analytes extracted by the SPME technique included DOC compounds with no known bioactivity as well as organic compounds known as biomarkers for specific vent-dwelling organisms. The percentage of biotic DOC was higher in Alba Vent, compared to the Urashima Ultranochichi samples.

Use of SPME under these extreme ecosystems could potentially help researchers identify specific organic compounds associated with active biological processes and better understand the ecology of vents and cycling of organic carbon.

## Results

**Figure 1:**
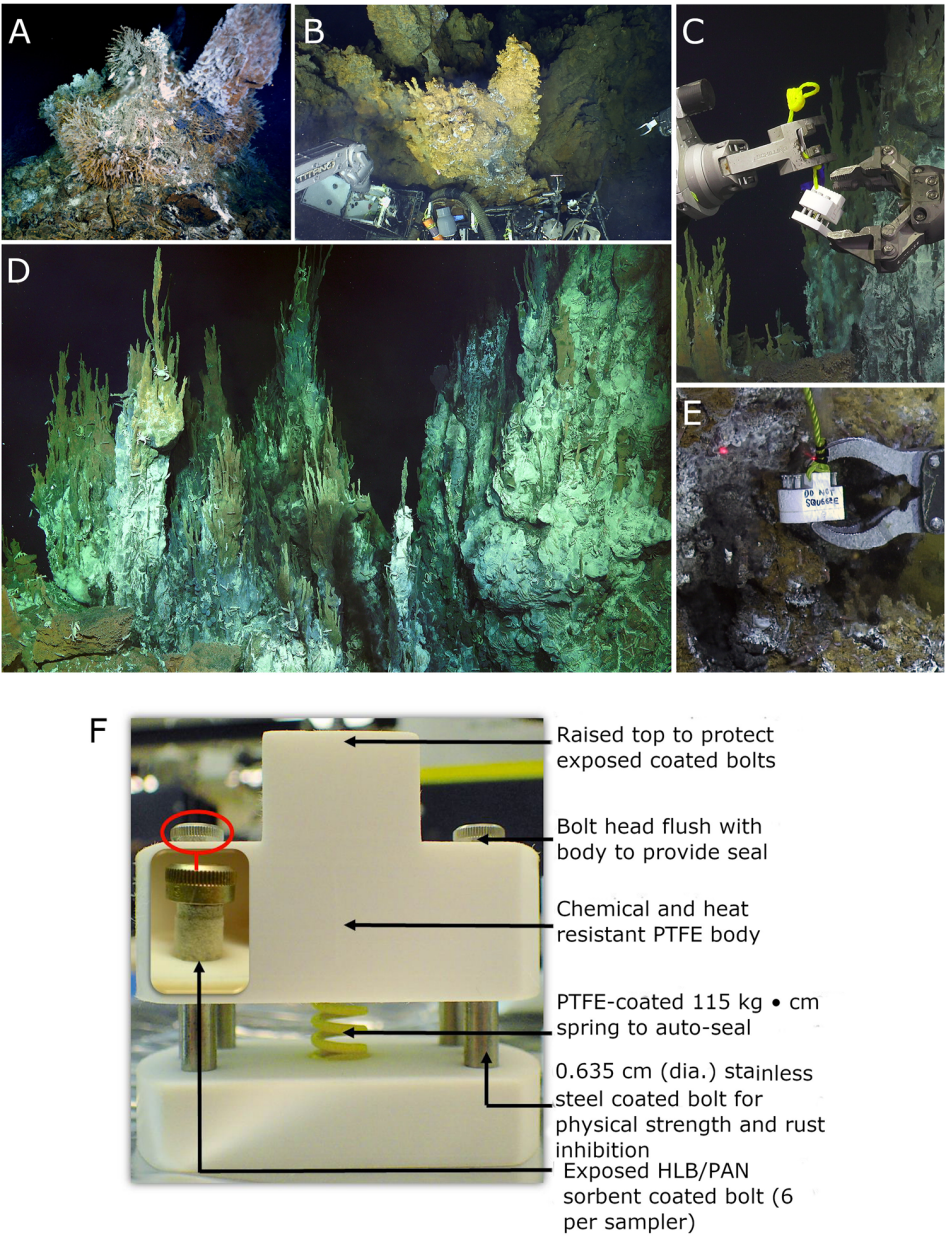
Sample locations and SPME *in situ*. Clockwise from upper left: **(A)** El Gordo chimney with tubeworms, Axial Seamount (Image credit: University of Washington); **(B)** Ultranochichi chimney, Urashima site, down-looking view of iron-oxide covered chimney (Image credit: Schmidt Ocean Institute); **(C)** SPME sampler manipulation at Alba vent (Image credit: Schmidt Ocean Institute); **(D)** Overview of Alba chimney complex (sample location in lower left), with shrimp and crabs. (**E)** Triggering of the SPME sampler at Ultranochichi. (**F**) Image showing details of the internal and external components of a coated bolt sampler used for sampling. (**A**): credit Univ. Washington; **(B**,**C)** credit Schmidt Ocean Institute.

**Figure 2:**
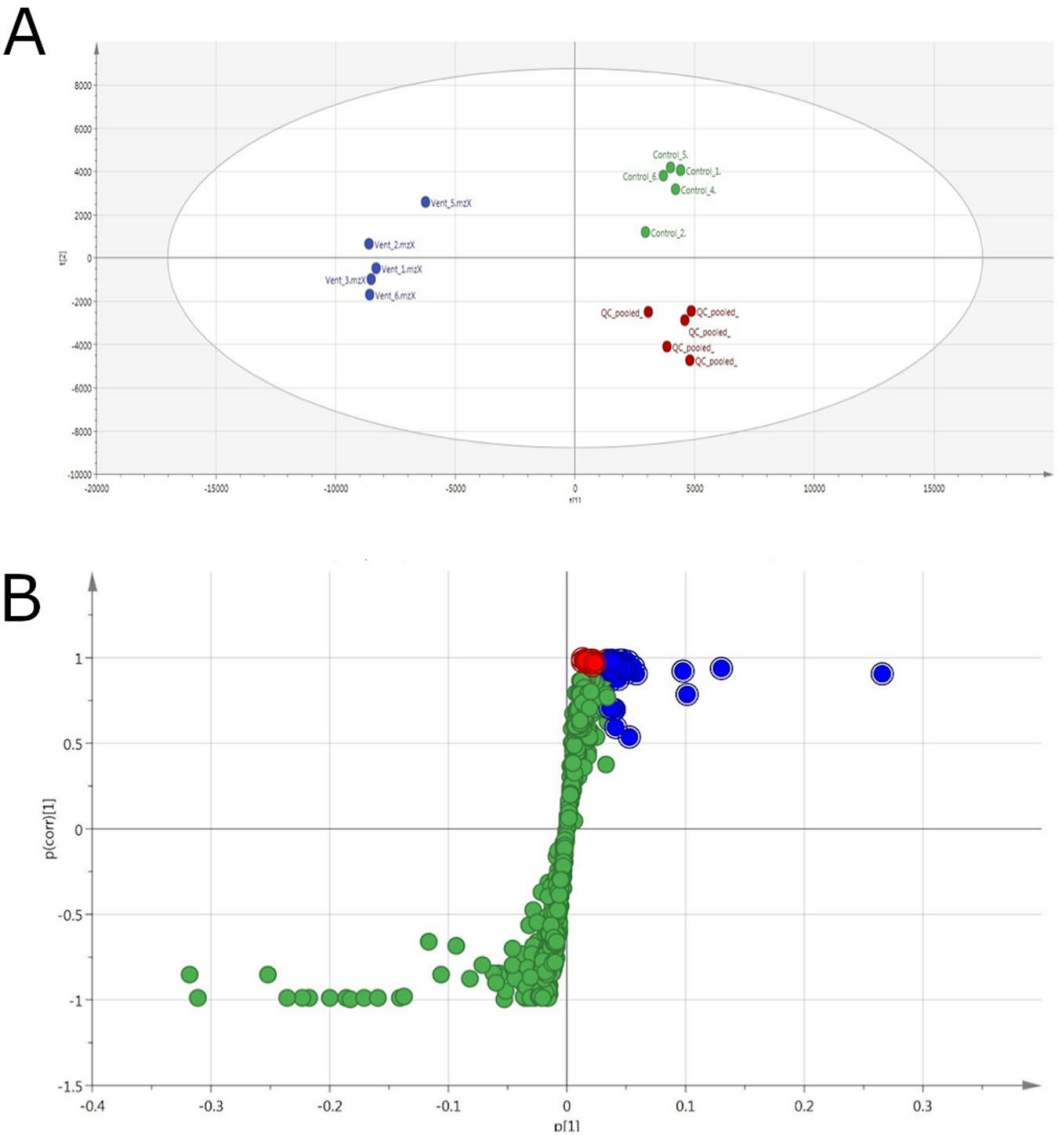
Multivariate data analysis information for the samples acquired from El Gordo hydrothermal vent. (**A**) Multivariate separation (PCA) of replicate samples corresponding to extractions from the El Gordo hydrothermal vent sample (BLUE), the background sample (GREEN), and the validating pooled QC data (RED). Despite the short 15-second sampling duration, excellent grouping and separation were observed for the samples. Samples were run on the PFP column and ionized in positive mode. Separation is based on the exact mass peak height. (**B**) S-Plot generated from the classed multivariate separation shown in section A, highlighting features in BLUE as statistically larger (VIP > 1) in the vent sample, and features in RED as exclusively present in the vent sample but with VIP < 1. GREEN highlights features that were commonly found among vent samples and controls, or in the controls only. An S-plot generated by SIMCA’s OPLS-DA statistical analysis model shows the correlation between variable magnitude (x axis) and correlation of the variable magnitude (y axis).

Multiple self-sealing SPME devices were prepared and deployed on three separate dives of submersible, remotely-operated vehicles (ROVs) for a broad metabolomics investigation of deep-sea hydrothermal vents. These samplers were designed to handle the extreme temperature and pressure present at these vents and were shown to be stable in temperatures exceeding 200 °C. The three vents sampled by SPME and an image showing the components of a self-sealing sampler are shown in Fig. 1. The first SPME sampler test was performed at the El Gordo vent, located at a depth of 1518 m at the Axial Seamount in the north-east Pacific Ocean. A map of the Axial Seamount on the Juan de Fuca Ridge can be seen in Figure S1. Unfortunately, due to a miscommunication between our joint team from the University of Waterloo, University of Victoria, and the ROV operator team, the sorbent coatings deployed in this mission were only exposed to the vent and background sample for a period of 15 seconds. Despite this incredibly short sampling time, it was pleasantly surprising to see excellent separation between the background and vent locations when unclassed principal component analysis was performed (Fig. 2A). This could be attributed to the large 250 mm^2^ surface area of the coated bolts; being 22.5 times larger than that of a classical SPME fiber, the response would theoretically be the same as if a 5.63 min fiber-based extraction was performed instead. This result highlights the importance of sampler design for efficient extraction of compounds in the environment. However, it is worth noting that only 5 of the 6 replicate samples from each site could be reliably plotted, as 2 of the non-recessed coatings were found to be damaged as a likely result of scraping on the sampler body. This incident prompted the development and subsequent use of recessed coatings on future dives.

After confirming reasonable unclassed separation of the samples, Orthogonal Projections to Latent Structures Discriminant Analysis (OPLS-DA) statistical processing was used to group samples. It is important to note that OPLS-DA is considered a classed multivariate approach that discriminates separations based on user-defined groups^16^. OPLS-DA is a supervised modeling approach where separation between groups is outlined. OPLS-DA was used to generate an S-plot and a related Variable Importance of Projection (VIP) list to differentiate significant features extracted from the samples. The S-plot tool was used for statistical analysis since it is able to unfold the contributions of the different sources to the distribution of a data sample in a given variable^17^. VIP values, on the other hand, were used to provide a summary of the overall contribution of each X-variable to the model, summed over all components and weighted according to the Y variation for each component^18^. Significant features were then selected using SIMCA software’s VIP value significance estimation, where m/z values assigned a VIP > 1 were considered differentially significant (Fig. 2B).

Most probably due to the short duration of the extraction carried out at the El Gordo vent zone, the number of identified compounds from this sample was relatively low in comparison to those attained from samples of the two other sites. The m/z values of the molecular ions were determined by Thermo Exactive MS, then used for compound predictions on the METLIN database and Sirius software. The list of predicted or verified analytes is presented in Table S1.

**Figure 3:**
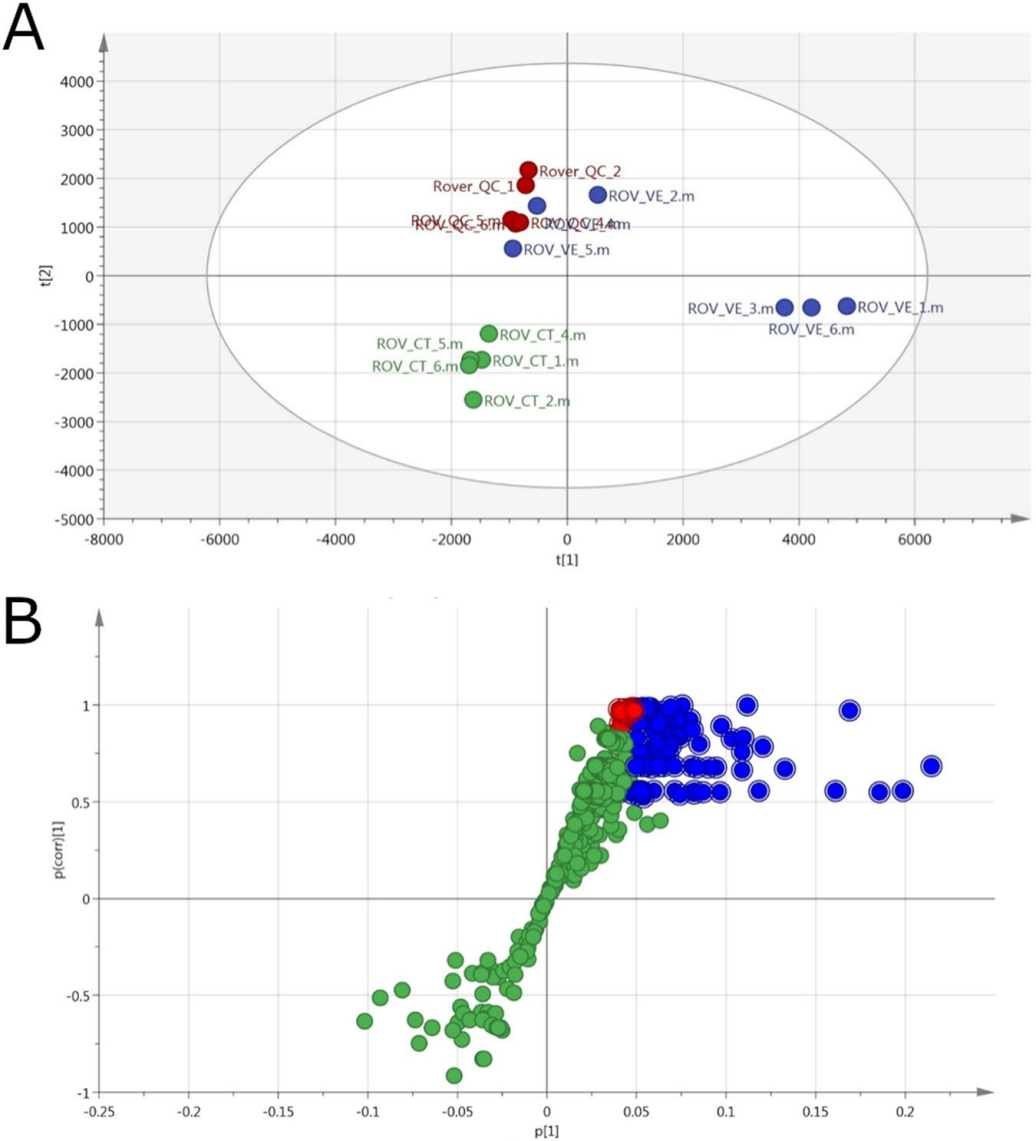
Multivariate data analysis information for the samples acquired from Ultranochichi vent. (**A**) Multivariate separation (PCA) of replicate samples corresponding to extractions from the Urashima Field hydrothermal vent sample (BLUE), the background sample (GREEN), and the validating pooled QC data (RED). Well grouped pool QC data indicates stable instrument performance. Samples were run on the PFP column and ionized in negative mode. Separation is based on the exact mass peak height. (**B**) S-Plot generated from the classed multivariate separation, highlighting features in BLUE as statistically larger (VIP > 1) in the Urashima vent sample, and features in RED as exclusively present in the vent sample but with VIP < 1. GREEN highlights features that were commonly found among vent samples and controls, or in the controls only.

A second SPME sampling was carried out at the Urashima vent site on the Mariana back-arc^19^, in an area of diffuse flow on the side of a sulfide chimney (Fig. 1) covered with an iron-rich mat^20^. The location of the chimney can be seen in the map provided in Figure S2. Extraction was carried out for 6 min, enabling the capture of a diverse range of compounds. A PCA plot (Fig. 3A) and an S-plot showing the VIP values (Fig. 3B) are presented.

The Ultranochichi vent sample yielded a greater number of analytes as compared to that from the El Gordo vent, though most analytes were observed in ESI-positive ionization mode. Analytes extracted from Ultranochichi vent ranged from low-MW organic compounds to high-MW organic compounds, consisting of mostly lipids (Table S2).

**Figure 4:**
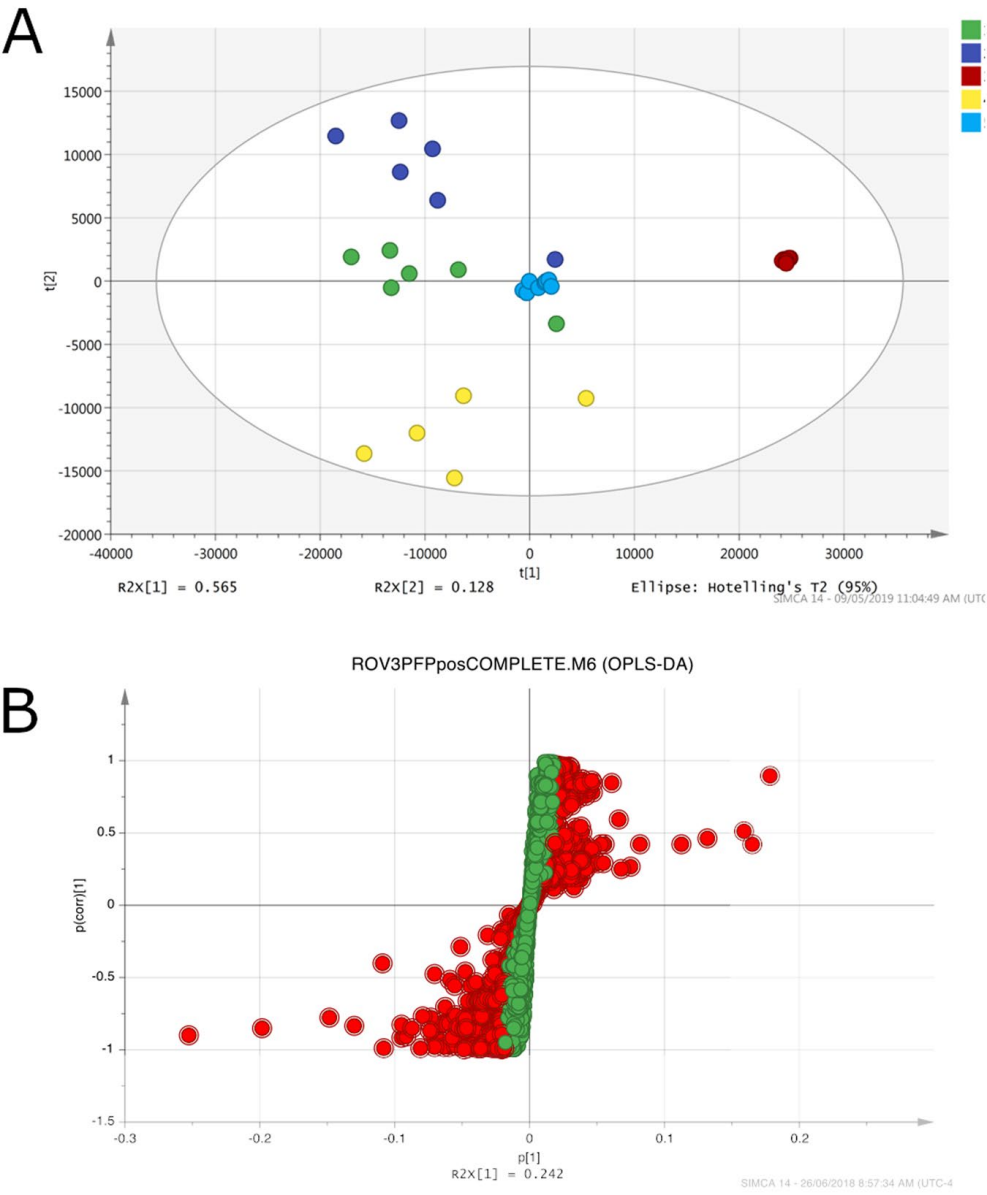
Multivariate data analysis information for the samples acquired from the Alba hydrothermal vent. (**A**) PCA plot of the 3rd ROV sampling, carried out at the Alba hydrothermal vent site along the Marina Back-arc, including both *in situ* and *ex situ* analysis results. PCA analysis of detected compound shows excellent unclassed separation and clustering of all sample types, and well clustered pooled QC data. Group 1 (green) represents *in situ* controls, group 2 represents *in situ* samplers (dark blue), group 3 represents *ex situ* samples (red), group 4 represents sampler blanks (yellow), and group 5 represents the pooled QCs (light blue). (**B**) S-plot generated from the OPLS-DA data, showing features with a VIP > 1 as RED for all samples.

A third *in situ* SPME sampling was carried out in a diffuse flow area in the Alba hydrothermal vent, located in the Hafa Adai vent field near 17 °N in the central Mariana back-arc (Figure S2) in December of 2016. This was the only site where water samples were collected for additional *ex situ* SPME analysis. This sampling yielded a very rich list of organic compounds, including a predominant number of unsaturated lipids. Among all three hydrothermal vents, the Alba vent hosted the most diverse biological community as determined by photographic imaging of the sites. PCA plots (Fig. 4A) show distinct separation of classes among vent samples and background samples. The generated S-plot supports the PCA results, showing significant differentiation among the two groups (Fig. 4B). A list of predicted compounds with their corresponding m/z values is presented in Table S3.

**Figure 5:**
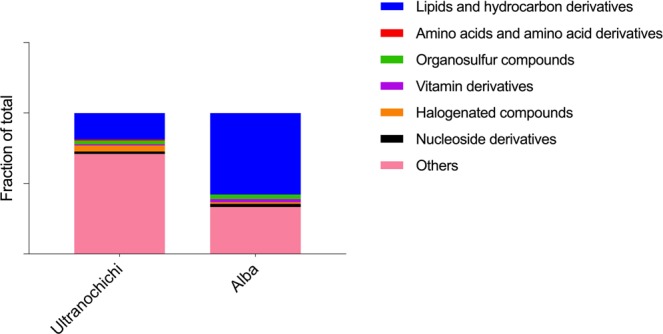
Stacked histogram showing the variability and diversity of organic compounds extracted via SPME from Ultranochichi and Alba vents. Alba vent compounds are comprised of a higher fraction of lipids and hydrocarbon derivatives. Features of El Gordo vent have not been included as the short sampling time does not allow for a true comparison of these sites.

Both the Ultranochichi and Alba vent samples yielded a wide range of compounds, including lipids and hydrocarbon derivatives, amino acids and their derivatives, organosulfur compounds, nucleoside and vitamin derivatives, and halogenated compounds. One notable finding was the wider range of lipids and hydrocarbon derivatives extracted from Alba vent as compared to Ultranochichi vent. A stacked histogram of identified compounds grouped into their biochemical and organic classifications (Fig. 5) shows that over half of the compounds extracted from the Alba vent were lipids or hydrocarbon derivatives.

El Gordo vent at the Axial Seamount and the Alba vent in the central Mariana back-arc support dense animal communities; conversely, fewer vent fauna were present on Ultranochichi chimney in the Urashima vent field. However, given that extraction at El Gordo was carried out for a significantly shorter period of time, a direct comparison of results from this location cannot be made with those taken from the Urashima or Alba vents. From the sample at Alba vent, we expected to see a contribution from the many animal species (limpets, crab, shrimp, etc.) living in the immediate vicinity of the sampling location. From Ultranochichi, the biotic contribution is presumed to primarily stem from the zeta-proteobacteria living in the thick, iron-rich microbial mats covering the outside of the chimney^20^ or from the thin white mat coating the active vent opening where the sample was taken. The vent fluids from Alba vent have elevated hydrogen sulfide and iron levels, producing a solid metal sulfide chimney favorable for animals. Conversely, Ultranochichi fluids are characterized by very low hydrogen sulfide levels and high iron levels that contribute to the observed fluffy iron-rich exterior of the sampled chimney.

**Figure 6:**
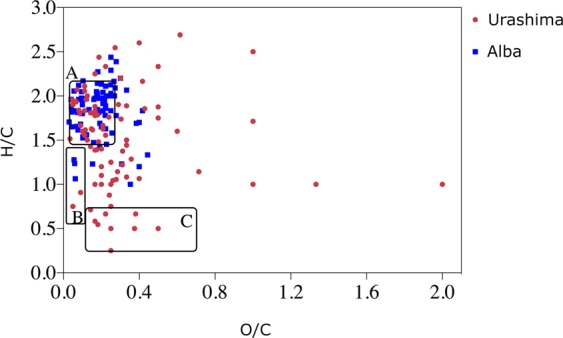
Van Krevelen diagram of organic compounds. These compounds were extracted from Ultranochichi (red) and Alba vents (blue). Clusters of Lipids (**A**), unsaturated hydrocarbons (**B**), and condensed hydrocarbons (**C**) are indicated in the diagram.

The nomenclature of the identified organic compounds along with their respective observed sites are given in Table S4. Furthermore, we have carried out a Van Krevelen diagram comparison to show the differences between the groups of organic compounds attained in these two hydrothermal vent samples. As can be seen in the Van Krevelen diagram, most of the compounds detected in the Alba vent were plotted in the zones specific to lipids and unsaturated hydrocarbons, while compounds detected in the Urashima field samples were characterized by a more disperse distribution, and included more condensed hydrocarbons (Fig. 6). The employed classification of compounds was adapted from Ohno *et al*.^21^.

The high H/C (≥1.3) and low O/C (≤0.48) ratios of DOCs were found to belong to highly-labile DOCs in a previous report by Rossel *et al*.^9^. Our findings within this range, which mostly consists of unsaturated hydrocarbons would be supporting the fact that SPME extracts a broad range of labile DOMs.

Our results also support the findings of Vorobev *et al*.^22^ where they showed that highly unsaturated dissolved organic matter (DOMs) and unsaturated aliphatic DOMs with low O/C ratios tend to be highly labile, compared to polycyclic aromatic and saturated DOMs.

In addition to *in situ* SPME sampling, water samples were also acquired from Alba vent and submitted to shipside TFME analysis (*ex situ*) followed by instrumental analysis at the University of Waterloo. Two groups of samples acquired from the same depth but at different temperatures (209 °C and 15 °C) (Tables S5 and S6) were compared with each other in terms of detected compounds in an effort to investigate significantly different features between these two samples. A comparison of predicted compounds attained from these samples and those attained from the sampling carried out *in situ* with the thin film coated bolts was also carried out, showing that the overall number of significant features attained via *ex situ* TFME was much lower than that attained via *in situ* sampling. Moreover, *in situ* sampling displayed a wide range of biologically important compounds, whereas *ex situ* sampling did not display such a diverse list of metabolites. This finding was most pronounced for lipids; *ex situ* sampling did not capture any lipids with known nomenclature, while the number of predicted lipids was very high for *in situ* sampling. We speculate this to be due to two reasons: (1) most labile DOCs were consumed by the microorganisms in the water samples, or (2) hydrophobic-hydrophobic interactions between the long hydrocarbon chains of lipids and the contact surfaces of the primary water samplers removed the lipids prior to *ex situ* TFME. This hydrophobic analyte loss has been explored in previous studies, finding that many compounds with a partition coefficient logarithm (Log*P*) >4.0 show major losses in any transferring vessel (e.g. pipette tips, vials etc.)^23^. These results greatly highlight one of the advantages associated with the use of *in situ* extractions via SPME for analysis of DOCs from species-rich ecosystems, namely the elimination of losses of hydrophobic compounds such as lipids.

Another possible reason for the observed differences between *in situ* vs. *ex situ* results concerns the possibility of losses related to labile DOMs. In SPME, once analytes are extracted onto the sorbent coating, they are chemically isolated from their previous aqueous matrix. This isolation should vastly decrease, or even eliminate, the rate of biochemical and oxidative changes, thus contributing towards a more accurate “snapshot” of the real sampling environment. Previous work by Vuckovic *et al*. demonstrated that some metabolites undergo oxidation once withdrawn from organisms; in that work, oxidized levels of glutathione were found to be elevated in blood samples withdrawn from mice as compared to levels attained via *in vivo* SPME. Similarly, in clinical studies on mice, β-NAD and adenosine monophosphate (AMP) detected by *in vivo* SPME could not be detected through exhaustive methods such as solvent precipitation and ultrafiltration^13,24^.

It is also important to note that while the TFME blades used for *ex situ* analysis possessed a much lower surface area as compared to that of the TFME bolts, *ex situ* extractions were performed for much longer (60 minutes vs. 12 minutes), although from a limited sample volume. However, considering that the blades have approximately half the surface area of the bolts, but were exposed to the sample for extraction for a period of time 6 times longer than that used in *in situ* extractions, we would have still suspected the blades to give more analyte signal assuming pre-equilibrium extraction. Lastly, environment temperature is assumed to play a large role in the differences observed between the two *ex situ* groups of samples. Likely due to the fact that a water temperature of 15 °C is more suitable for organisms to live in rather than 209 °C, more compounds were observed in the water sample gathered from the 15 °C hydrothermal vent site.

## Discussion

Deep sea hydrothermal vents are distinctly unique areas on the deep ocean floor; where many endemic species thrive^25^. Chemosynthesis, taking place through redox reactions among the host of compounds present in the vent fluids, supports the growth of diverse microorganisms that form the basis of both symbiotic relationships with animals and a food web of grazing metazoans. Organisms that thrive in these ecosystems have undergone distinct adaptations to overcome the stress caused by variations in temperature, pH, hydrostatic pressure, and especially, high levels of H_2_S, which are known to lead to the release or loss of endemic metabolites in non-adapted organisms. In the case of higher animals, high levels of H_2_S and hypoxia would cause problems on cytochrome-*c* oxidase systems. For example, alvinocaridid shrimps that live in their hydrothermal vent habitats have distinct mutations as positive selections on *atp6*, *cox1*, *cox3*, *cytb*, and *nad1–5* genes, which are involved in regulating the oxidative phosphorylation pathway^26^. One distinct adaptation of bacteria and archaea found in these ecosystems concerns the ability of such organisms to regulate their lipid composition in bilayer membranes to overcome these stresses^27^, which would potentially reflect to the free lipid composition in the surrounding waters.

There are several hypotheses on the source of DOCs in hydrothermal vent settings. Highly reducing conditions and surface areas of catalytic elements favour the synthesis of organic compounds from inorganic precursors^28,29^. These compounds, which are already synthesized by organisms, comprise amino acids^30^ and even long chain fatty acids^31^. However, the presence of a diverse range of biological species in these ecosystems and in the organic sediments found in such locations, which have been found to contain higher levels of biomarkers for microorganisms, C_24_–C_28_, and unsaturated fatty acids (as adaptations)^27^ in comparison to non-vent waters, could be another reason behind our observation of these fatty acids. However, the composition of gases and dissolved elements present in these systems in modern deep oceans would not be similar to the diversity of compounds that existed in the Archaean ocean^32^, mostly due to the development of an oxidative atmosphere and ocean. As an oxidative atmosphere and ocean developed, the habitat of hydrothermal vents changed accordingly. For this reason, biomarkers both from novel organisms or organic compounds that were related with the development of life can be displayed in these ecosystems.

SPME is a non-exhaustive analytical technique that minimizes the impact on the biological system being sampled^33^. SPME does not modify the environment, i.e. through the emission of radiation or introduction of foreign compounds. This minimally invasive method, in contrast to exhaustive methods — especially the ones which require the removal of organism samples from the site — does not cause significant chemical or biological effects on the environment. With our TF-SPME coated bolt samplers, minute amounts of compounds released from organisms through chemical or biological means were extracted in these deep-sea environments. In this regard, the sampler design presented here is both simple and effective; three ROV teams were able to easily execute the deployments using simple instructions.

Further, while many labile DOCs and DOSs are unlikely to remain in either fluids or organisms brought to the surface for sampling^34^, the protein-resistant coating of the SPME device renders metabolites released by organisms nonlabile to degradation or modification by enzymes^34^. Even though the very short durations of the *in situ* extractions carried out in this work were likely not sufficiently long so as to allow most analytes to reach equilibrium with the sorbent material, a high amount of biomarker compounds was nonetheless successfully extracted. In this regard, given the equilibrium-based extractive nature of SPME, substantially longer durations of SPME would not necessarily extract correspondingly larger amounts of free analytes, since a compound will cease to diffuse onto the coating once equilibrium between the phase and sampling environment is reached.

Among the extensive number of compounds extracted either *in situ* or *ex situ* via SPME, numerous compounds were identified with the use of tentative annotation, standard verification on high-resolution tandem MS (stated as ST in the Tables), online databases, and the Sirius software. These compounds were mainly, but not limited to: amino acids, lipids, hydrocarbons, organosulfurs, thiol-, benzene-, and phenol-bearing compounds. The large variety of detected organosulfur compounds may reflect the abundance of dissolved sulfur species in hydrothermal vent fluids. A Van Krevelen plot drawn to facilitate a comparison between Alba and Ultranochichi vents shows significantly more clustering of samples from Alba vent within the zone for “lipids” as compared to samples from Ultranochichi, which yielded a more diverse distribution of organic compounds, including both saturated and unsaturated hydrocarbons. Higher abundance and diversity of lipids at the Alba site may be associated with the dense animal communities present at the Alba sampling site, in contrast to the iron-rich mat with few animals at the Ultranochichi chimney sampling site.

Likely due to the short duration of the extraction carried out at El Gordo vent, which lasted less than a minute, only compounds with molecular weights of less than 500 Da could be extracted. Compounds with larger MWs would not have had time to diffuse to the sorbent coating, since the diffusion coefficient of compounds increases with increasing molecular weight^35^. Among the compounds extracted in this site, one notable finding entailed the detection of sulfur-bearing organic compounds such as S-methylcysteine sulfoxide, 3-sulfinoalanine, and S-methyl methanesulfinothioate. The compound 3-sulfinoalanine is a sulfonated amino acid involved in cysteine and methionine metabolism^36^. However, this compound is also known to take part in taurine metabolism as a precursor of hypotaurine, which functions in some vent animals to form thiotaurine to inhibit the toxicity of H_2_S^37^ and is also a very labile DOS^7^. The tubeworm *Ridgeia piscesae*, commonly found in northeast Pacific hydrothermal sites including El Gordo, forms dense clumps where it transfers dissolved sulphide to its symbionts. This animal is known to bear high levels of thiotaurine in its symbiont-bearing tissues, especially in deep sea locations where fluid supply delivers a high sulphide flux^38^.

S-methyl methanesulfinothioate, another significant compound detected at El Gordo, is a freely reactive compound known to be involved in the thiol group to dithiomethane group (-S-S-CH_3_) modification^39^. The presence of this compound in hydrothermal vent ecosystems has not been reported before. We postulate that this compound may be related with the synthesis and modification of organosulfur compounds.

Notably, a number of sulfur-bearing long chain fatty acids (sulfolipids) were extracted from the Mariana back-arc vent fluids. The presence of sulfur in the molecular structures of detected compounds such as Leukotriene E4 methyl ester, 2-(Octadecyloxy)thiophene, and N-(3E-hexadecenoyl)-deoxysphing-4-enine-1-sulfonate, suggests that the high concentrations of sulfur in these ecosystems may contribute to the formation of these organic compounds in animals. Sulfur is known to replace phosphate to form sulfolipids (such as N-(3E-hexadecenoyl)-deoxysphing-4-enine-1-sulfonate), which highly decrease phosphorus demand by picocyanobacteria in oligotrophic marine environments^40^.

Our LC-MS/MS data included a large variety of monounsaturated and polyunsaturated fatty acids. Bacterial strains isolated from shallow water environments are characterized by the predominant presence of hydrocarbons with chain lengths of less than 20^41^. However, bacteria that live in high pressure environments, known as barophiles, have adapted the composition of their membrane lipids by increasing their lipid carbon chain length and the unsaturation of these lipids^42^. These polyunsaturated fatty acids include docosahexaeneoic acid and eicosapentaenoic acid^43,44^. Although only tentatively verified, our results predict the existence of either microorganisms or animals capable of synthesizing hydrocarbons with very high chain lengths (more than 20) (See Table S2 and S3 formulas) in Ultranochichi and Alba vent. We believe that the identified phospholipids with odd-numbered, higher hydrocarbon chain lengths (e.g., C21, C23) likely originated from microorganisms^45,46^, or eukaryotic sphingosines^47^. These lipids correspond to mono-or polyunsaturated PEs, PCs, and PAs, which are known to be present on both prokaryotic and eukaryotic cell membranes.

Hydrothermal vent animals also contain polyunsaturated fatty acids (PUFAs) in their cellular membranes. These compounds are believed to play crucial roles in the stability and functionality of the cellular membranes of these organisms^48^. Many holobionts transfer PUFAs from chemoautotrophic bacteria to the host, as is the case of both *Ridgeia piscesae*^49^ and the hairy snail species *Alviniconcha* found at Alba vent. As PUFAs are not available at hydrothermal vents, some animals appear to synthesize them^48^ in a manner similar to certain non-vent molluscs, which have species-specific fatty acyl desaturases and elongases to lengthen the hydrocarbon chains of unsaturated fatty acids^50,51^. Similarly, cephalopods and crustaceans were found to synthesize unsaturated fatty acids. The branchiopod *Daphnia pulex* is one such organism to show increased production of PUFAs once exposed to low temperatures, even though it is believed to strictly feed on a PUFA-free diet^52^. DNA sequencing data has shown that the limpet *Lottia gigantean* possesses genes that have homology to coding sequences of putative elongases with potential functions in PUFA synthesis^53^.

Another interesting finding was the presence of sterols among the list of analytes, which would correspond to the presence of fungi in these environments. Of note, an isomer of ergosterol (a sterol with 29 carbon atoms and one hydroxyl group) was detected among the extracted analytes. As ergosterols are lipids predominantly synthesized by fungal species, we can consider them to be biomarkers for fungi in the habitat. The presence of this isomer of ergosterol (m/z 413.3783) among the samples could thus potentially indicate the presence of a deep-sea fungus species. Yeast from the genus *Rhodotolula* has been previously isolated in Mid Atlantic Ridge vents at 2300 m depth^54^. The presence of fungi can be further supported by the presence of a possible sterol with two additional hydroxyl groups (m/z 437.3248 at Alba vent). These sterols, alternatively called “triols”, have been recognized in yeasts, fungi, and deep-sea sponges. For example, the marine-derived fungus *Trichoderma* sp. can synthesize cholesta-7,22-diene-3β,5α,6β-triol^55^. The analogous compounds of this triol showed enzyme inhibition towards *Taq* DNA polymerase and HIV polymerase^56–58^. Although our technique cannot verify the biological source of the extracted sterols, it is possible that this triol was a metabolite of a hydrothermal vent fungus species.

Ladderane fatty acids were among some of the tentatively annotated hydrocarbons in our list of identified compounds. As reported by Russ *et al*., ladderane fatty acids are biomarkers for anaerobic ammonium oxidation (anammox) bacteria, which oxidize ammonium to reduce nitrite to N_2_^59^_._ DNA materials related to anammox bacteria and ladderane lipids have been isolated from several Atlantic hydrothermal vents^60^. Our data show that ladderane fatty acids were present in both Ultranochichi and Alba vent. While ladderane fatty acids are not stable at high temperatures ranging from 120 °C to 365 °C^61^, low temperature spots (17–15 °C) in these locations may support the presence of anammox bacteria.

Even though they live in the darkness of the deep ocean, vent animals need vitamin D for regulation of their calcium metabolism^62^. The Ultranochichi sample contained (6R)-6,19-epidioxy-1α-hydroxy-6,19-dihydrovitamin D3, a vitamin D3 derivative, which we believe to be a metabolically active form of vitamin D3, as dihydrovitamin D3 is metabolically active. For most marine organisms, phytoplankton is the source of this vitamin; it is delivered to the deep sea via migrating zooplankton and fish or sinking organics. In addition, the early life stages (larvae) of some organisms may rise to the surface and feed on zooplankton enriched with this vitamin during development, as described for a hydrothermal vent shrimp^63^ closely related to the species under our samplers in the Mariana region. The amount of vitamin D precursors in zooplankton can reach 50 μg/g dry weight^64^.

Tetrahydroxycholestenoic acids were present among the compounds extracted from the Alba vent (m/z 465.319). These compounds are metabolic products of cholesterol, which is a biomarker of eukaryotic cells. Cholesterol is the dominant sterol in bythograeid crabs and vent squat lobsters at East Pacific Rise vent^65^ – species of the same genera were very close to our sampler, as was *Rimicaris*, a shrimp for which cholesterol is known to be present in an allied species^66^.

In summary, the *in situ* implementation of SPME in hydrothermal vent zones was demonstrated to yield superior results as compared to *ex situ* SPME experiments carried out shipside, even though the durations of the three separate extractions carried out *in situ* were much shorter than that of *ex situ* experiments. Recently, our group demonstrated an alternative approach to green sampling, where the thin film extraction phase is placed directly in a water grab sampling vessel such as a bottle or jar. This approach, termed “in-bottle TFME”, overcomes potential losses of compounds during sampling and transportation while allowing for standard grab sampling to be performed^67^. Considering that deploying ROVs and keeping the TF-SPME sampler stable at a specific spot of a chimney for long periods of time (e.g. 60 min.) is tedious and expensive, the use of in-bottle TF-SPME devices could be further investigated as a potential sampling method for investigations under the extreme conditions found in hydrothermal vents. In addition to the already discussed benefits imparted by the use of *in situ* SPME, this approach could potentially enable the acquisition of higher numbers of samples in a relatively inexpensive manner as compared to ROV deployment. Future studies are needed to assess the suitability of the in-bottle SPME approach for longer extraction periods, which would enable the diffusion of compounds to reach equilibrium, and thus facilitate quantitation of compounds present in these environments.

## Conclusion

In this work, novel coated bolt TF-SPME samplers were demonstrated to successfully extract a wide range of analytes from diffuse hydrothermal vents, as well as enable sample differentiation between vent samples and samples attained in distant control areas of the deep ocean. Detected analytes included a range of DOCs; some known as biomarkers for different genera of organisms and are distinct within variable hydrothermal vent plumes. The results of this work corroborate the suitability of SPME as a simple, efficient, and inexpensive method for the extraction of labile DOMs and can potentially be used for the annotation and quantification of labile compounds that have various biological activities. Our results support that organisms synthesize highly unsaturated lipids under extreme pressures. Even though light cannot reach these deep-sea hydrothermal vents, the presence of a vitamin D3 derivative indicates that some organisms thriving in these ecosystems may potentially consume other organisms that carry out photosynthesis. The *in situ* sampling approach was shown to generate a far greater number of significant features as compared to the *ex situ* TFME method used in this work, demonstrating the great potential of *in situ* SPME methods to deliver significantly more impactful and informative representations of such complex sampling environments in future studies.

## Materials and Methods

### Chemicals and materials

HPLC-MS grade water, acetonitrile, and methanol were obtained from Fisher Sci. Canada (Ontario, Canada). Formic acid, ammonium acetate, 37% hydrochloric acid, dimethylformamide, 150 kDalton polyacrylonitrile (PAN) were purchased from Sigma-Aldrich (St. Louise, Missouri, USA). The rare earth magnets were purchased from Lee Valley Tools (Waterloo ON, Canada). The 18-8 stainless steel springs, nuts, and bolts were purchased from Spaenaur (Kitchener, ON, Canada). Swagelok model 177-R3A-K1-B PTFE-coated springs were purchased from Swagelock Inc. (Sarnia, ON, Canada). The PTFE sampler bodies were constructed at the Science Machine Shop, University of Waterloo (Waterloo ON, Canada). The 5 µm and 30 µm, hydrophilic−lipophilic balanced (HLB) particles, which were used as functional particles in the coatings of the devices, were obtained by Waters (Wilmslow, U.K.)

### Preparation of the coated bolt SPME devices

The first coated bolts were prepared using a spray coating methodology adapted from a procedure first reported by Musteata *et al*. and advanced by Mirnaghi *et al*.^68,69^. These bolts were used for sampling from the El Gordo vent. Briefly, this procedure entailed first dissolving 150 kDa polyacrylonitrile (PAN) in dimethylformamide (DMF) at a 10% PAN weight percentage, and then mixing 10 mL of the resulting solution with 1.0 g of 30 µm HLB particles and 3 mL of DMF so as to prepare a sprayable slurry. The surfaces of the stainless-steel bolts were etched by hanging the coatable surface of each bolt in an open beaker of concentrated HCl under sonication for 10 minutes. An Aldrich glass sprayer (Sigma-Aldrich, Oakville, ON, Canada) was used to apply approximately 10–12 coats of the slurry. Each coat was set in a modified GC oven at 150 °C. These coated bolts were then cleaned and conditioned in a 50:50 methanol: water solution.

For bolts prepared with a recessed extraction phase, which were used in the Urashima Field and Alba vent samplings, etching was performed for 1.5 hours, which resulted in a 30 µm indentation on the stainless-steel surface. Dip coating was then performed using a programmable actuator such that the bolts could be immersed in the aforementioned PAN/HLB/DMF slurry up to the edge of the etched surface. Furthermore, a smaller and more strongly sorbing 5 µm HLB particle was used for preparation of these coatings. The dip coating method entailed application of two coats of the slurry, with each coat being set in a modified GC oven at 150 °C. Thereafter, any excess coating was removed from the head of the bolt using a utility knife, whereupon the coating was cleaned and conditioned in a 50:50 methanol: water solution.

HLB/PAN TFME blades were prepared by dissolving PAN in DMF and preparing HLB/PAN slurries in the above concentrations for spray-coating on stainless steel blades, as described before by Mirnaghi *et al*.^68^.

### Field sampling

We conducted three experiments to test the effectiveness of the self-sealing coated bolt sampler design for sampling at hydrothermal vent zones. Sampling was conducted in vent locations characterized by low temperatures and diffuse fluids where vent animals were present. In each case, a ‘control’ sampler puck was carried to the seafloor but not deployed, while a third puck was deployed to detect background signals in deep-sea water. Although three different Remotely Operated Vehicles (ROV) conducted the deployments, the sampler design was easily adapted to each operator. The coated bolts of the SPME “puck” were shielded during transit to the seafloor in a sample box. On site, the puck was lifted from the box by one manipulator and carefully positioned by a second manipulator within the claws of the former; only when the sampler was in position in the venting fluid did the claws squeeze to expose the bolts. With the manipulator locked, the bolts remained stationary. Following sampling, the puck was placed in a closable ROV sample box for the remainder of the dive and ascent. Once on board, the devices were stored at −80 °C for the remainder of the voyage, then shipped on dry-ice to the University of Waterloo for desorption and analysis. Table 1 presents the locations and specifics of each deployment.

**Table 1:** Site information for samples taken from vents on two sides of the Pacific Ocean.

Site (vent name, vent field or site)	Location	ROV	Depth (m)	Temperature (°C)	Sample Type	Duration (min)	Notes
El Gordo, International District	Axial Volcano, Juan de Fuca Ridge	ROPOS	1520	63	SPME	0.25	Sampler above tubeworms in cooler water
Above vent, International District	Axial Volcano, Juan de Fuca Ridge	ROPOS	1518	~2	SPME	0.25	Sampler about 2 m above vent
Ultranochichi, Urashima Field	Mariana Backarc Spreading Centre	Jason-2	2928	17	SPME	6.4	Vent with iron deposits and few animals
Background, Urashima Field	Mariana Backarc Spreading Centre	Jason-2	~2750	1.5	SPME	6	Rising at end of dive
Alba Vent, Hafa Adai	Mariana Backarc Spreading Centre	Subastion	3277	15	SPME	12	Sampler among shrimp and crabs
Background, Perseverance	Mariana Backarc Spreading Centre	Subastion	~3890	1.5	SPME	10	Rising from seafloor at end of dive
Alba Vent, Hafa Adai	Mariana Backarc Spreading Centre	Subastion	3277	15	TFME	50	Water sampled from same vent
Alba Vent, Hafa Adai	Mariana Backarc Spreading Centre	Subastion	3277	209	TFME	60	Water sampled from black smoker

Vent site information is listed in the Interridge Vent Database 3.4*. Duration describes the lengths of time the thin-film coated bolts were exposed to vent fluid.

* https://vents-data.interridge.org/.

The experiment at the El Gordo vent on Axial Volcano in the northeast Pacific was a pilot test for compatibility with ROV operations. As the vent deployment time was inadequate (15 s) and the “background” sampler was too close to the vent site, results are not likely to be comprehensive. This sample was taken on a small chimney mound with a high temperature spout (Fig. 1A). Sampling took place over a bush of tubeworms (*Ridgeia piscesae*). Although the temperature at the base of the bush was over 60 °C, the puck was positioned for sampling in cooler fluid, just above the tubeworms. The background seawater sampler was opened at about 2 m above the mound.

In December 2014, we were able to sample a small orifice on a chimney in the southern Mariana Back-arc Spreading Centre, located in the northwest Pacific. The Ultranochichi vent in the Urashima field is a multi-spired iron-rich edifice with a spigot venting at about 180 °C. In this location, sampling was carried out in fluid emerging on the side of the chimney at a temperature of approximately 17 °C (Fig. 1B,C). Animal abundance was relatively low in this location, with vent shrimp and crabs clustered around the diffuse flow where a white microbial mat was visible. Due to time constraints, the background sampler was held open after the ROV left the vent to ascend; thus, background sampling was carried out at a depth range of 2830 to 2670 m for six minutes.

The Alba vent, located in a newly discovered vent site in the central trough of the Mariana back-arc^70,71^, comprises a complex structure of massive sulphide cones, blocky rubble, and thin spires rising about 6 m (Fig. 1D). Fluid emerges from several places, including from smokers with temperatures measured at 238 °C. The SPME puck was deployed in a 16 °C vent near the base of the structure. Observed vent animals included barnacles, limpets, shrimp, and crabs. The background sample in this region had to be collected on another dive, in a vent field located approximately 150 km to the south; this circumstance was not ideal but, at depths over 3000 m in this oligotrophic ocean, the ambient water is likely to be very similar. Fluids were also collected from the same site on the Alba vent for *ex situ* analysis (Table 1). For this purpose, a Hydrothermal Fluid and Particle Sampler was used for “cold water” sampling to slowly pump fluid through a Teflon and titanium manifold until all hoses were filled with vent water^72^. In addition, the high flow-rate smoker was sampled with a spring-loaded titanium 750-mL syringe “major” sampler for collection of “hot water” samples. On the ship, 8 mL aliquots of each fluid were partitioned into 10 mL vials, from which *ex situ* extractions were performed using an HLB/PAN thin film blade for 60 minutes at 1000 rpm. After extraction, the sorbent coating of these blades was stored in a 2 mL vial at −80 °C and shipped to University of Waterloo.

### Desorption of large surface area coated screw device

As described previously by Grandy^73^, desorptions were carried out by placing the coated bolts in a narrow, high-density polyethylene (HDPE) centrifuge tube. Only the coated side of the bolt was immersed in the solvent. 800 µL of 50:50 acetonitrile:water was used to desorb each of the devices. The tubes were vortexed and then agitated at 1200 rpm for 75 minutes. Following desorption, the solutions were then aliquoted into 2 mL amber glass vials for storage and analysis. Pooled QC’s were prepared by aliquoting 100 µL of solution from each individual sample. All of the aqueous desorption solutions were further stored at −80 °C.

### Instrumental and data-processing analysis method (High-resolution HPLC-MS)

As described previously by Grandy^73^, the analytical instrumentation used for the separation and detection of the analytes was a Thermo Acella autosampler-HPLC and an Exactive Orbitrap MS (Thermo Fisher Scientific, San Jose, California, USA). Chromatographic separations were performed using a Supelco Discovery pentafluorophenyl (PFP) HS F5 column with dimensions 2.1 mm × 100 mm, 3 μm (Supelco, Bellefonte, PA, USA). For positive mode electrospray ionization (ESI), gradient elution was performed using a 2-component system that consisted of mobile phase A (99.9:0.1% water: formic acid v:v) and mobile phase B (99.9:0.1% acetonitrile: formic acid v:v) and for negative mode ESI, mobile phase A (99:1 water: ammonium acetate buffer (20 mM) v:v) and mobile phase B (9:1 acetonitrile: ammonium acetate buffer (20 mM) v:v). The flow rate was 300 μL min^−1^ at all times. Initial mobile phase conditions were 100% A (0–3.0 min), followed by a linear gradient to 10% A from (3.0–25.0 min), and an isocratic hold at 10% A until 34.0 min. The total run time was 40 min per sample, with a 6 min column re-equilibration period. The gradient elutions for the HPLC method are represented in Figure S3.

**Figure 7:**
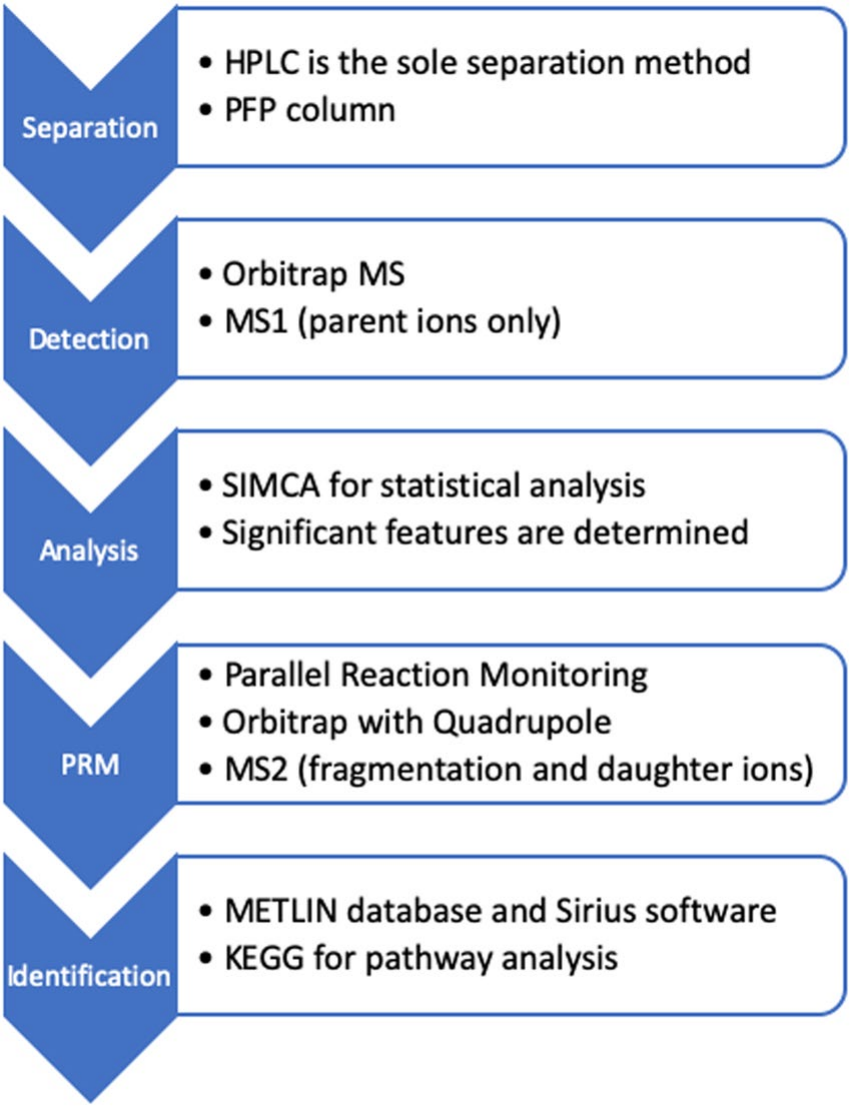
Process scheme for analysis and identification of analytes.

As presented in Figure 7, analyses were performed in positive and negative ESI modes using the described PFP-HPLC method. The injection volume for both of the methods was 10 μL and the storage temperature was 4 °C on the autosampler. The samples were analyzed in a randomized order. Instrument QC’s and pooled QC’s were run regularly to verify instrument performance. MS acquisition was performed using AGC = balanced (1,000,000 ions) with a 50,000 resolution at 2 Hz. The injection time onto the C-trap was 100 ms. Sheath gas (arbitrary units (AU)), auxiliary gas (AU), sweep gas (AU), ESI voltage (kV), capillary temperature (°C), and vaporizer temperature (°C) were 30, 10, 5, 4.0 (−2.9 negative mode), 300, and 300. The acquisition range was 100–1000 m/z. External instrument mass accuracy calibration was performed every 48 hours and ensured that it was within 2 ppm. This HPLC-MS metabolomics procedure was adapted from the methodology previously reported by Vuckovic^74^.

Data processing was performed by initially converting the.raw data files to an mzXML format using the MSconvert software^75^. The converted files were then imported into the software MZmine 2^76^. Using a Savitzky-Golay filter (5 data point), scan-by-scan filtering was performed on the imported data. A mass peak list was generated using exact mass detection, m/z range of 99–1000 m/z, and mass tolerance of 5.0 ppm. Chromatogram builder settings were: minimum peak height of 10,000 AU and a minimum width of 0.017 min. Deconvolution of these rebuilt chromatograms was performed using a Savitzky-Golay filter with a minimum peak height of 10,000 AU and peak width setting of 0.017–1.0 min. The peak list was filtered using an m/z-range of 99–1000, a retention time range of 0.8–35 min and peak width of 0.017–1.0 min. SIMCA-14 multivariate data processing software was further used to process the peak lists. Principal component analysis (PCA) was performed using Pareto scaling for determination of significant features and to test the statistical fit of the data acquired from sample analysis. Significant features were identified by the METLIN database within a 5 ppm mass tolerance^77^.

Chromatographic separation and tandem MS analysis were carried out on the QCs of each experimental sample and on a single mixture of standard compounds (100 ppb each) with the use of a high-resolution Q-Exactive Thermo Orbitrap MS (Thermo Fisher Scientific, San Jose, California, USA) operated under the same parameters as described above (same column, methodology, and mobile phases) for samples. MS acquisition was performed through particle reaction monitoring (PRM), with a resolution of 70,000, isolation window of 1.6 m/z, normalized collision energy (dimensionless) of 20 eV and 40 eV, using automatic gain control (AGC) balanced (1,000,000 ions) at 2 Hz. The injection time onto the C-trap was 100 ms. Instrumental settings were: Sheath gas (35 AU), auxiliary gas (5 AU), sweep gas (300 AU), capillary temperature (280 °C), and vaporizer temperature were 35, 5, 300, and 280 °C and the acquisition range was 70–1000 m/z, for the positive and negative ESI methods. External instrument mass calibration was performed every 48 h and was ensured to be within 2 ppm. PRM was carried out on a list of MS1 m/z values that corresponded to all m/z values of VIP > 1, which were determined by the SIMCA software.

As the last step, raw data files of the LC-MS/MS data were imported on MZmine. Chromatograms corresponding to each MS1 m/z on target retention time points were exported as.PRM files. The exported.PRM files were further imported to Sirius 4.0.1 software with settings corresponding to the specific ESI mode and MS2. Feature computations were performed by Sirius using the following software settings: Orbitrap, 5 ppm, 10 candidates, and all PubChem formulas. The predictions of the software were only taken into consideration if at least 3 possible peaks were predicted. A CSI:FingerID search^78^ was performed and the results were compared with the MS1 METLIN annotations. Additional to the fragmentation pathway analysis, we used the Fragment Similarity function of the METLIN database by inputting the highest 4 peaks on the MS2 chromatograms. In cases where METLIN database’s fragment/daughter ion spectra data were relevant with the groups on MS1-predicted compounds, we chose the compound to be our final annotation. For cases where METLIN database’s fragment prediction was not sufficient, we used the molecular formula prediction of the Sirius software as our final annotation. As examples, the fragmentation mass spectra of parent ions 221.0962, 419.3159, and 440.3575 are given in Figures S4, S6, S8, and the fragmentation trees are given in Figures S5, S7, S9, respectively. In cases where the use of standards of compounds for identification was feasible, we compared the fragmentation patterns of the compounds on their specific retention time points. If the fragmentation patterns were the same, the compound was considered to be verified, and no further analysis of spectra was carried out on METLIN or Sirius.

### Determination of compound classifications

Compounds were classified according to their roles in the hydrothermal vent ecosystems. Organosulfur compounds, fatty acids, amino acids, vitamins, nucleosides, and halogenated compounds were reported in different groups. Organic compounds were specified in 4 subgroups based on their elemental structure (CHO, CHOS, CHNO, CHNOS). Furthermore, Van Krevelen plots of elemental ratios of H/C and O/C were plotted to determine the variability of detected organic carbon sources.

### Statistical analysis of features by SIMCA

We used SIMCA software for statistical analysis of features determined using the above described method. Principle Components Analysis (PCA), which is a mathematical algorithm that reduces the dimensionality of the data while retaining most of the variation in the data set^79^, and Orthogonal Projections to Latent Structures Discriminant Analysis (OPLS-DA) statistical processing were used to group samples. It is important to note that OPLS-DA is considered a classed multivariate approach that discriminates separations based on user-defined groups^16^. It is therefore important to highlight that mild separation between groups was still observed when unclassed principle component analysis (PCA) was performed.

In terms of feature selection, a feature loading S-plot that separates features based on the OPLS-DA separation was first prepared. From this S-plot, features found to separate at the top right and bottom left of the main linear cluster were manually selected and listed in a table. Features in this list were furthered filtered with the use of SIMCA’s proprietary Variable Importance in Projection (VIP) algorithm, where features possessing a VIP >1.000 were deemed significant^80^. It should be highlighted, however, that VIP has been deemed a “black box” algorithm by some users given that details pertaining to VIP have been kept secret by Umetrics^80^. Moreover, it has been previously indicated that VIP filtering may unnecessarily remove features that exhibit lower relative signals even if they reveal an absence-presence relationship between 2 samples. Nonetheless, a selection of significant features differentiating the biochemical profiles of two samples extracted by the coated bolts was successfully achieved through the use of the described methodology.

## Supplementary information

Supplementary Information.

## Data Availability

The data can be found in the online repository: 10.5281/zenodo.3462526.
