# Supplementary material for: Unique Solid Phase Microextraction Sampler Reveals Distinctive Biogeochemical Profiles among Various Deep-Sea Hydrothermal Vents
Source: Sci Rep. 2020 Jan 28;10:1360. doi: 10.1038/s41598-020-58418-4 (PMC6987176; doi:10.1038/s41598-020-58418-4)
Supplement: Supplementary file 1 — Supplementary Information. [file 41598_2020_58418_MOESM1_ESM.pdf]

Supporting Information for

Unique Solid Phase Microextraction Sampler Reveals Distinctive Biogeochemical Profiles among Various Deep-Sea Hydrothermal Vents

Jonathan James Grandy[1†], Bora Onat[1†], Verena Tunnicliffe[2], David Butterfield[3,4], Janusz Pawliszyn[1]

[1]Department of Chemistry, University of Waterloo, 200 University Avenue West, Waterloo, ON, Canada
[2]Department of Biology, University of Victoria, Victoria, BC, Canada
[3]NOAA/PMEL, 7600 Sand Pt Way, Seattle, Washington, NE, 98115-6349, USA
[4]JISAO, University of Washington, Washington, 98115-6349, USA

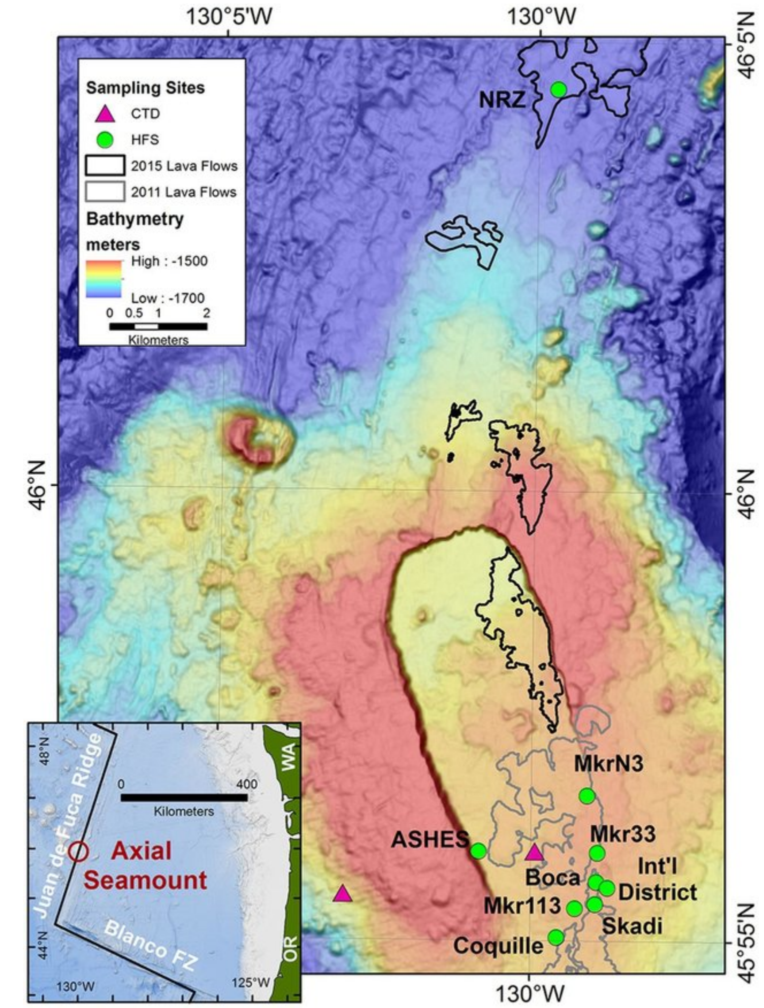

Figure S1. Map of Axial Seamount on the Juan de Fuca ridge. Hydrothermal fluid samples were collected for this study at the International District vent field in the SE caldera, at the El Gordo sulfide chimney. Map from Topcuoglu et al., 2016, Frontiers in Microbiology[1]. Images were generated in ArcGIS v10.7[2].

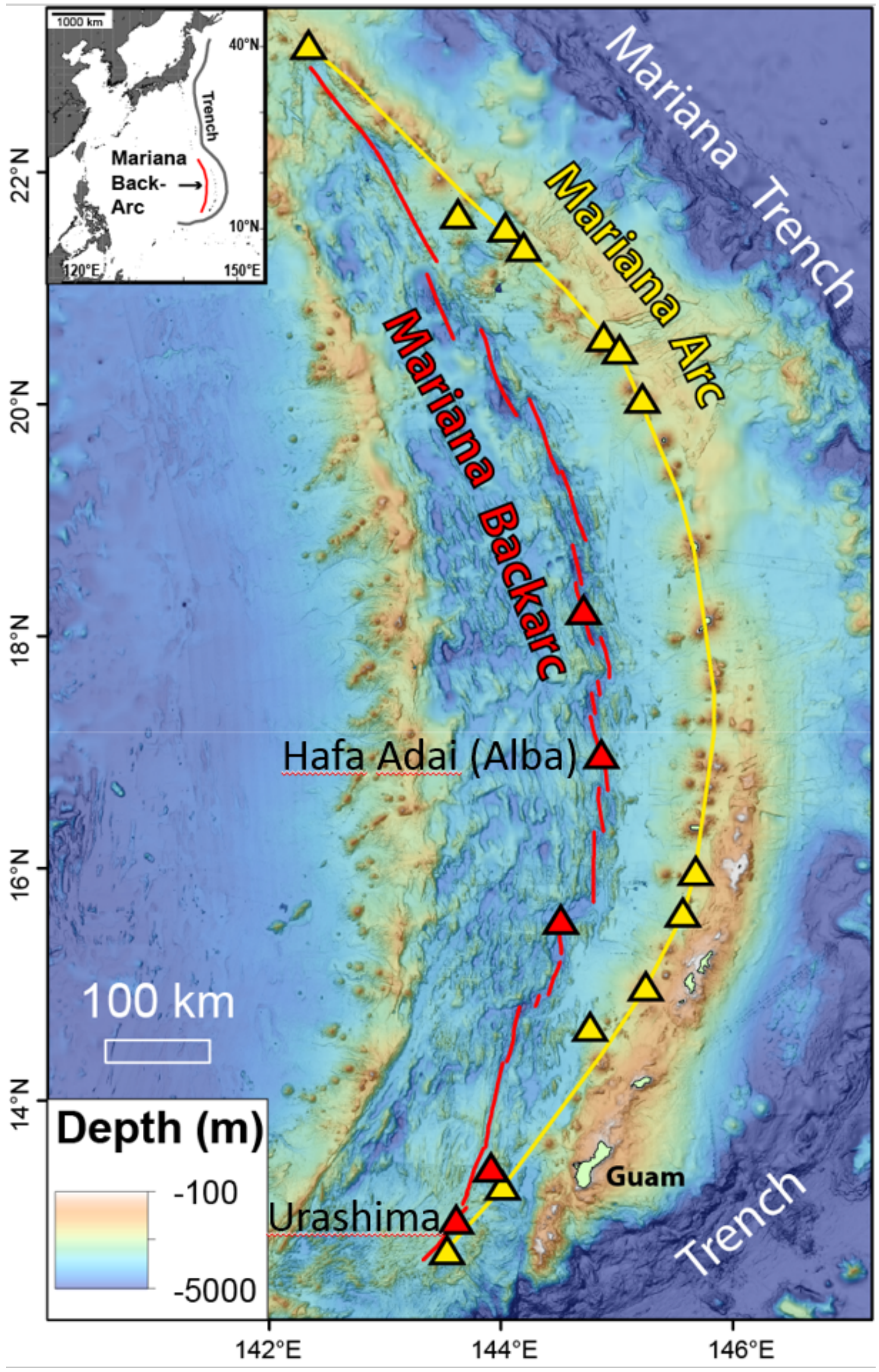

Figure S2. Map of Mariana Region, showing active hydrothermal sites on the arc (yellow) and back-arc (red). Samples were collected in 2014 at the Urashima site, and in 2016 at the Alba chimney, located in the Hafa Adai vent field. Map produced by W. Chadwick, from Butterfield et al., submitted May 2019[3]. Images were generated in ArcGIS v10.7[2].

Table S1. Classification of identified organic compounds according to their biochemical importance.

Chemical name	Formula	m/z value	Adducts	Sampling Site
Lipids and hydrocarbon derivatives				
Equilin sulfate	$C_{18}H_{20}O_5S$	197.041	$[M+H+Na]^{2+}$	Urashima
Leukotriene E4 methyl ester	$C_{24}H_{39}NO_5S$	418.2432	$[M+H-2H_2O]^+$	Urashima
Palmitic acid	$C_{16}H_{32}O_2$	257.977	$[M+H]^+$	Urashima
1-tetradecanyl-2-(8-[3]-ladderane-octanyl)-sn-glycerol	$C_{37}H_{68}O_3$	281.2664	$[M + 2H_2]^{2+}$	Urashima
1-(15-methyl-tridecanyl)-2-(8-[3]-ladderane-octanyl)-sn-glycerol		281.2664	$[M + 2H_2]^{2+}$	Urashima
A fatty acyl amide	$C_{14}H_{29}NO$	227.9549	$[M+H]^+$	Urashima
A hydrocarbon derivative	$C_{21}H_{38}N_6O_3$	423.1987	$[M+H]^+$	Urashima
N-cyclohexylnonanamide	$C_{15}H_{29}NO$	239.9664	$[M+H]^+$	Urashima
(4E,8E,10E-d18:3)sphingosine	$C_{18}H_{33}NO_2$	318.2398	$[M+Na]^+$	Urashima
A fatty acyl amide	$C_{18}H_{32}O_3$	297.2612	$[M+H]^+$	Urashima
A sphinganine	$C_{25}H_{49}N_{13}O_3$	580.4152	$[M+Na]^+$	Urashima
Docosenamide	$C_{22}H_{43}NO$	338.3413	$[M+H]^+$	Urashima
A diglyceride (DG)	$C_{22}H_{34}$	299.2769	$[M+2H]^{2+}$	Urashima
	$C_{42}H_{79}NO_7S$	780.5178	$[M+H]^+$	Urashima
A fatty acyl amide	$C_{12}H_{27}NO_2$	219.1064	$[M+H]^+$	Urashima
A C19 sphingolipid analog	$C_{20}H_{38}N_2O$	323.3052	$[M+H]^+$	Urashima
A type of decanediamide	$C_{28}H_{50}N_6O_5$	551.3942	$[M+H]^+$	Urashima
A C18 fatty acyl amide	$C_{18}H_{37}NO_2$	282.2834	$[M+H]^+$	Urashima
9,9-dibromo-8-nonenoic acid	$C_9H_{14}Br_2O_2$	312.9426	$[M+H]^+$	Urashima
Nonane- or heptanedioate	$C_{13}H_{22}O_5$	259.1541	$[M+H]^+$	Alba
3-hydroxy-dodecanedioic acid	$C_{12}H_{22}O_5$	269.1385	$[M+Na]^+$	Alba
Linoleamide	$C_{18}H_{33}NO$	280.2636	$[M+H]^+$	Alba
Stearamide	$C_{18}H_{37}NO$	284.2949	$[M+H]^+$	Alba
Dihydroxy-hexadecanoic acid	$C_{16}H_{32}O_4$	306.2639	$[M+NH_3]^+$	Alba
Dihydroxy-palmitic acid	$C_{16}H_{32}O_4$	306.2639	$[M+NH_3]^+$	Alba
Eicosadienoic acid	$C_{20}H_{36}O_2$	309.2791	$[M+H]^+$	Alba
An octodecenol acetate or eicosanoic acid	$C_{20}H_{38}O_2$	311.2948	$[M+H]^+$	Alba
	$C_{17}H_{21}N_5O$	312.1828	$[M+H]^+$	Alba
	$C_{18}H_{34}N_3O_4$	357.2612	$[M+H-H_2O]^+$	Alba
A MG	$C_{23}H_{42}O_4$	383.314	$[M+H]^+$	Alba
5S-HETE di-endoperoxide	$C_{20}H_{34}O_8$	404.2362	$[M+H]^+$	Alba

Ergosterol	$C_{28}H_{44}O$	413.3783	$[M+H]^+$	Alba
$C_{24}H_{46}O_5$	$C_{24}H_{46}O_5$	415.3431	$[M+H]^+$	Alba
An heptanoic acid	$C_{21}H_{30}N_4O_4$	425.2147	$[M+H]^+$	Alba
	$C_{24}H_{44}O_6$	429.319	$[M+H]^+$	Alba
	$C_{25}H_{49}N_3O$	430.3765	$[M+H]^+$	Alba
	$C_{24}H_{48}O_6$	433.3524	$[M+H]^+$	Alba
	$C_{26}H_{44}O_5$	437.3248	$[M+H]^+$	Alba
	$C_{20}H_{40}N_6O_5$	445.3137	$[M+H]^+$	Alba
A type of tetrahydroxycholestenoic acid	$C_{27}H_{44}O_6$	465.319	$[M+H]^+$	Alba
A hexanoic acid or a methylbutanoic acid	$C_{22}H_{44}N_6O_5$	473.345	$[M+H]^+$	Alba
	$C_{25}H_{53}N_5O_2$	478.4103	$[M+Na]^+$	Alba
Nb-Lignoceroyltryptamine	$C_{34}H_{58}N_2O$	549.4198	$[M+K]^+$	Alba
A 30-carbon PC	$C_{30}H_{64}NO_6P$	566.4536	$[M+H]^+$	Alba
DG	$C_{39}H_{60}O_4$	593.4543	$[M+H]^+$	Alba
Ceramide	$C_{38}H_{75}NO_3$	610.4797	$[M+K]^+$	Alba
DG	$C_{41}H_{64}O_5$	637.4809	$[M+H]^+$	Alba
1-(9,14-dimethyl-pentadecanoyl-2-(8-[3]-ladderane-octanyl)-sn-glycerol	$C_{40}H_{72}O_4$	655.5089	$[M+K]^+$	Alba
DG	$C_{36}H_{67}O_8P$	659.4615	$[M+H]^+$	Alba
PE-Cer(d14:2/19:0)	$C_{37}H_{73}N_2O_7P$	667.4818	$[M+Na]^+$	Alba
	$C_{33}H_{65}N_{11}O_4$	680.5312	$[M+H]^+$	Alba
Sphingoyelin or spingodienine	$C_{37}H_{73}N_2O_7P$	689.5237	$[M+H]^+$	Alba
PC or PE (monounsaturated)	$C_{37}H_{72}NO_8P$	690.5067	$[M+H]^+$	Alba
Ergosteryl oleic acid	$C_{46}H_{76}O_2$	699.5468	$[M+H]^+$	Alba
1-tetrahexanoyl-2-(8-[3]-ladderane-octanyl)-sn-glycerophosphoethanolamine or a PE	$C_{39}H_{72}NO_7P$	715.5395	$[M+NH4]^+$	Alba
Bacteriohopane-31,32,33,34-tetrol-35-cyclitol	$C_{41}H_{73}NO_9$	723.5255	$[M]^+$	Alba
PE	$C_{41}H_{74}NO_7P$	724.5288	$[M+H]^+$	Alba
PE	$C_{39}H_{79}N_2O_7P$	741.5552	$[M+Na]^+$	Alba
PC or PE	$C_{40}H_{82}NO_7P$	742.5695	$[M+Na]^+$	Alba
MGDG	$C_{43}H_{68}O_{11}$	761.4816	$[M+H]^+$	Alba
A type of PE (monounsaturated)	$C_{43}H_{78}NO_8P$	768.5476	$[M+H]^+$	Alba
A type of PE (4 unsaturations)	$C_{43}H_{78}NO_8P$	768.555	$[M+H]^+$	Alba
PA(P-20:0/19:0)	$C_{42}H_{83}O_7P$	769.551	$[M+K]^+$	Alba
A type of PE (4 unsaturations)	$C_{43}H_{78}NO_8P$	785.5813	$[M+NH4]^+$	Alba
PS	$C_{43}H_{84}NO_9P$	812.5759	$[M+Na]^+$	Alba
PC	$C_{52}H_{96}NO_7P$	916.6565	$[M+K]^+$	Alba

A TG (12 unsaturations)	$C_{59}H_{90}O_6$	917.66	$[M+Na]^+$	Alba
Amino acids and amino acid derivatives				
S-methylcysteine sulfoxide	$C_4H_9NO_3S$	152.0363	$[M+H]^+$	El Gordo
3-Sulfinoalanine	$C_3H_7NO_4S$	171.0437	$[M+NH4]^+$	El Gordo
5-amino-2-(methylaminomethylcarbamoyl)-5-oxopentanoic acid	$C_8H_{15}N_3O_4$	216.9505	$[M+H]^+$	Urashima
Other organosulfur compounds				
S-Methyl methanesulfinothioate	$C_2H_6OS_2$	128.0195	$[M+NH4]^+$	El Gordo
Sulfanylethanamine or a methanesulfonamide	$C_3H_9NO_2S_2$	156.0144	$[M+H]^+$	El Gordo
Methyluric acid or propanesulfinothioate	$C_6H_6N_4O_3$, $C_6H_{12}OS_2$	147.0309	$[M+H-2H_2O]^+$	El Gordo
3-Methylcyclohexanethiol	$C_7H_{14}S$	169.0456	$[M+K]^+$	Urashima
Benzothiazolone	C_7H_5NOS	152.0362	$[M+H]^+$	Urashima
S-methyl Isothiourea	$C_2H_6N_2S$	134.9958	$[M-H + 2Na]^+$	Urashima
	$C_{10}H_{22}N_2O_3S$	251.1431	$[M+H]^+$	Alba
2-(Octadecyloxy)thiophene	$C_{22}H_{40}OS$	335.2792	$[M+H-H_2O]^+$	Alba
A sulfanylnaphthalene or naphthalene thiol	$C_{26}H_{40}S$	385.2925	$[M+H]^+$	Alba
Vitamin derivatives				
(6R)-6,19-epidioxy-1α-hydroxy-6,19-dihydrovitamin D3	$C_{27}H_{44}O_4$	433.3313	$[M+H]^+$	Urashima
(5Z)-1,25-dihydroxy-3-thiavitamin D3 / (5Z)-1,25-dihydroxy-3-thiacholecalciferol	$C_{26}H_{42}O_2S$	401.2873	$[M+H-H_2O]^+$	Alba
A cholecalciferol	$C_{29}H_{48}O_2$	429.373	$[M+H]^+$	Alba
Halogenated compounds				
	$C_8H_3FO_3$	167.0131	$[M+H]^+$	El Gordo
A difluoro indanone	$C_9H_6F_2O$	169.0461	$[M+H]^+$	El Gordo
	$C_6H_{14}BNO_3$	159.1379	$[M+H]^+$	Urashima
	$C_{10}H_{11}BrO_2$	272.9591	$[M-H]^-$	Urashima
A bromophenyl compound		274.9567	$[M-H]^-$	Urashima
	$C_{20}H_{15}Br_2N_3OS$	501.921	$[M-H]^-$	Urashima
	$C_{12}H_{12}BrN_3O_3S_2$	387.9402	$[M-H]^-$	Urashima
	$C_{17}H_{17}FN_2O_6$	365.1149	$[M+H]^+$	Alba
Nucleoside derivatives				
Pyrizine or pyrimidine methylphosphonic acid	$C_5H_7N_2O_3P$	175.0258	$[M+H]^+$	El Gordo
6-Methylthioguanine	$C_6H_7N_5S$	146.0299	$[M+H-2H_2O]^+$	Urashima

A type of diamino pyridine	$C_5H_7N_3$	148.0275	$[M+K]^+$	Urashima

Table S2. Annotated compounds listed by ascending order of m/z value. Analytes were extracted *ex situ* by TFME from samples attained from a high flow-rate smoker (209°C) in the Alba hydrothermal vent site with the use of a spring-loaded titanium 750-mL syringe "major" sampler. M, METLIN database MS/MS annotation; S, SIRIUS software's prediction.

m/z	Retention time (min.)	VIP-value	ESI mode (+/-)	Adducts	Annotation	Identification method
130.0169	3.45	2.68035	+	$[M+K]^+$	N'N-dimethyl-1-phosphanylmethanamine	S
146.0299	3.23	2.00026	+	$[M+H]^+$	1-(nitromethyl)-2H-tetrazol-5-one	S
151.0089	3.21	3.15166	+	$[M+H]^+$	N,N''-dinitro-urea	S
151.0353	3.45	8.80895	+	$[M+2Na]^{2+}$	$C_{12}H_{16}O_6$	S
157.0152	3.42	1.75075	+	$[M+H]^+$	Benzyldisulfide	S
165.0609	3.44	1.28697	+	$[M+H]^+$	1-nitro-1,3,5-triazinane-2,4-diol	S
171.0677	3.42	3.29205	+	$[M+H]^+$	1-diaminophosphoryl-2-methylbenzene	M, S
172.0638	3.45	2.93019	+	$[M+Na]^+$	$C_5H_{13}N_2OS$	S
176.0224	3.43	1.42086	+	$[M+K]^+$	N-5-pyrimidinylacetamide	M, S
182.9853	16.26	1.16708	+	$[M+H]^+$	$C_5H_6Cl_2NO_2$	S
187.0816	3.43	1.98139	+	$[M+H]^+$	L-arginine, nitrosated	S
188.0769	3.45	1.55204	+	$[M+H]^+$	$C_5H_9N_5O_3$	S
189.0973	3.46	1.93771	+	$[M+H]^+$	L-asparaginnyl-glycinamide	M, S
193.0559	3.45	1.36648	+	$[M+H]^+$	$C_{10}H_{10}NOS$	S
194.0594	3.44	1.35328	+	$[M+H]^+$	4-azidobutylmetahnesulfonate	M, S
198.042	3.45	1.68264	+	$[M+H]^+$	$C_7H_8N_3O_2P$	S
205.0922	3.44	1.10157	+	$[M+H]^+$	N'-Nitro-N-nitroso-N-pentylurea	M, S
205.0922	3.44	1.10157	+	$[M+H]^+$	omega nitro-arginine	M, S
208.0395	16.26	1.22187	+	$[M+K]^+$	$C_{11}H_9N_2$	S
218.9838	3.42	2.21105	+	$[M+H]^+$	$C_5H_4ClN_4O_2P$	S
227.9829	1.22	1.09196	+	$[M+K]^+$	(4-aminophenyl)phosphate	M, S

Table S3. Annotated compounds listed by ascending order of m/z value. Analytes were extracted *ex situ* by TFME from samples attained at the Alba hydrothermal vent (15°C) with the use of a Hydrothermal Fluid and Particle Sampler (HFPS). M, METLIN database MS/MS annotation; S, SIRIUS software's prediction.

m/z	Retention time (min.)	VIP-value	ESI mode (+/-)	Adducts	Annotation	Identification method
106.0043	3.45	1.36496	+	$[M+H]^+$	cyanomethyl phosphinic acid	S
128.0194	3.25	6.05778	+	$[M+K]^+$	1-methyl-3-methylideneurea	M, S
130.0169	3.24	2.68035	+	$[M+H]^+$	2-chloropyrimidine-5-amine	M, S
146.0299	3.23	2.00026	+	$[M+Na]^+$	$C_2H_7N_2O_4$	S
148.0274	3.24	1.13112	+	$[M+H]^+$	1-isocyanatophosphanyl-3-methylurea	S
148.0512	3.26	1.18878	+	$[M+H]^+$	pyrido[3,4-b]pyrazin-7-ol	S
149.0479	3.24	1.23469	+	$[M+H]^+$	bis(aziridin-1-yl)phosphinic acid	S
149.0597	13.1	1.53527	+	$[M+H]^+$	cinnamate	M, S
149.0597	13.1	1.53527	+	$[M+H]^+$	chromanone	M, S
150.0631	19.25	1.62944	+	$[M+H]^+$	$C_4H_9N_2O_4$	S
151.0089	3.45	2.152	+	$[M+Na]^+$	CH_6NO_6	S
151.0308	3.22	1.04349	+	$[M+H]^+$	C_8H_7OP	S
152.0097	3.21	4.49805	+	$[M+H]^+$	$C_7H_3O_4$	S
153.0328	3.25	4.80934	+	$[M+H]^+$	methylsufamoyl acetamide	M, S
153.0394	3.26	1.04986	+	$[M+H]^+$	S-n-butyl methanethiosulfinate	S
158.0118	3.28	1.3656	+	$[M+K]^+$	pyrimidine acetonitrile	M, S
158.0118	3.28	1.3656	+	$[M+K]^+$	Methylpyrimidine carbonitrile	M, S
162.0074	3.2	1.21868	+	$[M+K]^+$	(2-aminoethyl)methyl phosphinic acid	M, S
163.0754	14.63	1.3576	+	$[M+H]^+$	2-phenylbutyrolactone	M, S
170.0412	3.44	1.22573	+	$[M+H]^+$	$C_7H_8BClO_2$	S
170.0663	3.29	4.07286	+	$[M+H]^+$	$C_5H_7N_5O_2$	S
171.0435	3.26	1.01339	+	$[M+H]^+$	$C_{11}H_6O_2$	S
171.0677	3.22	1.03447	+	$[M+H]^+$	1-diaminophosphoroyl-2-methylbenzene	M, S
172.0638	3.24	2.09451	+	$[M+H]^+$	P-(3-aminophenyl)phosphonic diamide	M, S
177.0427	3.22	1.12084	+	$[M+K]^+$	$C_3H_{13}N_3OP$	S
183.0615	3.21	1.01434	+	$[M+H]^+$	Diphenylsilane	S

187.0816	3.24	1.39302	+	$[M+H]^+$	L-arginine, nitrosated	S
193.0635	3.26	1.02217	+	$[M+H]^+$	A short peptide	S
197.0409	3.25	5.1525	+	$[M+H]^+$	3-(2-aminoethyldisulfanyl)-L-alanine	M, S
200.0769	3.25	1.11227	+	$[M+H]^+$	N'-Hydroxy-3-(2-nitro-1H-imidazol-1-yl)propanimidamide	M, S
200.9725	3.25	1.12976	+	$[M+H]^+$	Dimethylimidazolidine-2-selone-4,5-dione	S
203.0766	3.26	1.96547	+	$[M+H]^+$	2-methyl-5-(phenylsulfanylmethyl)pyrrole	S
205.0922	3.21	1.61948	+	$[M+H]^+$	N2-nitro-N-nitroso-N-pentylurea	M, S
212.0944	3.26	1.12265	+	$[M+H]^+$	7-hydroxyheptyl sulfamate	S
213.0904	3.26	1.29491	+	$[M+H]^+$	Methylpropanyl naphthalenediol	M, S
217.0725	3.24	1.38411	+	$[M+H]^+$	Azobis(pyridine N-oxide)	M, S
219.9844	3.24	1.53074	+	$[M+H]^+$	$C_6H_8NO_2P_3$	S
220.9813	3.23	1.67707	+	$[M+Na]^+$	Phosphoenol-4-deoxy-3-tetrulosonate	M, S
223.9885	3.24	2.28432	+	$[M+H]^+$	$C_{12}H_2NO_2P$	S
226.9516	1.84	9.16997	+	$[M+K]^+$	3-fluoro-2-phosphonooxylpropanoic acid	S
228.1082	3.25	3.30359	+	$[M+H]^+$	$C_8H_{13}N_5O_3$	M, S
230.1057	3.26	1.16713	+	$[M+H]^+$	1,3,7-triaza-5-phosphabicyclononane, 3,7-diacetyl-	S
230.1238	3.27	1.6892	+	$[M+H]^+$	$C_8H_{15}N_5O_3$	S
233.0871	3.24	1.11739	+	$[M+H]^+$	Hydrazinyl methylidene glycyl-D-aspartic acid	M, S
235.1027	3.24	1.10985	+	$[M+H]^+$	$C_7H_{14}N_4O_5$	S
241.9997	3.21	2.65936	+	$[M+H]^+$	$C_{12}H_4NO_3P$	S
243.9973	3.22	1.22556	+	$[M+H]^+$	2-Bromophenylalanine	M, S
247.1391	3.25	1.08647	+	$[M+H]^+$	A small peptide	M, S
261.0306	3.24	1.885	+	$[M+H]^+$	2-amino-4-(S-methyl-N-phosphonosulfonimidoyl)butanoic acid	S
263.0282	3.21	1.02017	+	$[M+K]^+$	$C_6H_{12}N_2O_7$	S
265.0157	3.24	2.37545	+	$[M+K]^+$	$C_8H_{15}Cl_3O_3$	S
267.0132	3.22	1.73025	+	$[M+H]^+$	2-(4-bromoanilino)-N-prop-2-ynylacetamide	M, S
280.9932	3.2	1.39514	+	$[M+H]^+$	Cycloheptatrien-1-one derivative	M, S
284.0466	3.22	324.0466	+	$[M+H]^+$	$C_{16}H_{10}ClNO_2$	M, S
300.0241	3.18	1.28454	+	$[M+H]^+$	$C_{12}H_{14}BrNO_3$	M, S
305.1809	3.27	1.28616	+	$[M+H]^+$	A short peptide	M, S
315.1227	20.26	3.48591	+	$[M+H]^+$	2'-Hydroxyenterolactone	M
316.1261	19.37	1.10407	+	$[M+H]^+$	$C_{17}H_{15}F_2N_3O$	S
338.108	19.37	1.40083	+	$[M+H]^+$	$C_{12}H_{19}NO_{10}$	S
342.0885	3.39	1.20723	+	$[M+H]^+$	$C_{20}H_{11}N_3O_3$	S
344.1574	20.33	1.08157	+	$[M+H]^+$	$C_{13}H_{21}N_5O_6$	S

365.136	20.83	5.49381	+	$[M+H]^+$	$C_{17}H_{20}N_2O_7$	M, S
366.1393	20.87	1.15683	+	$[M+H]^+$	$C_{14}H_{23}NO_{10}$	S
430.9138	1.88	3.25298	+	$[M+K]^+$	D&C Red No.30	M, S

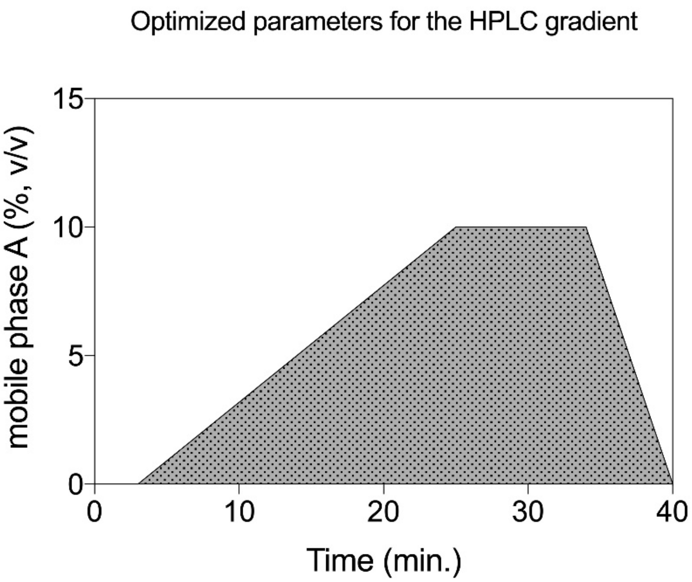

Figure S3. Optimized parameters for the HPLC gradient used for high resolution LC-MS.

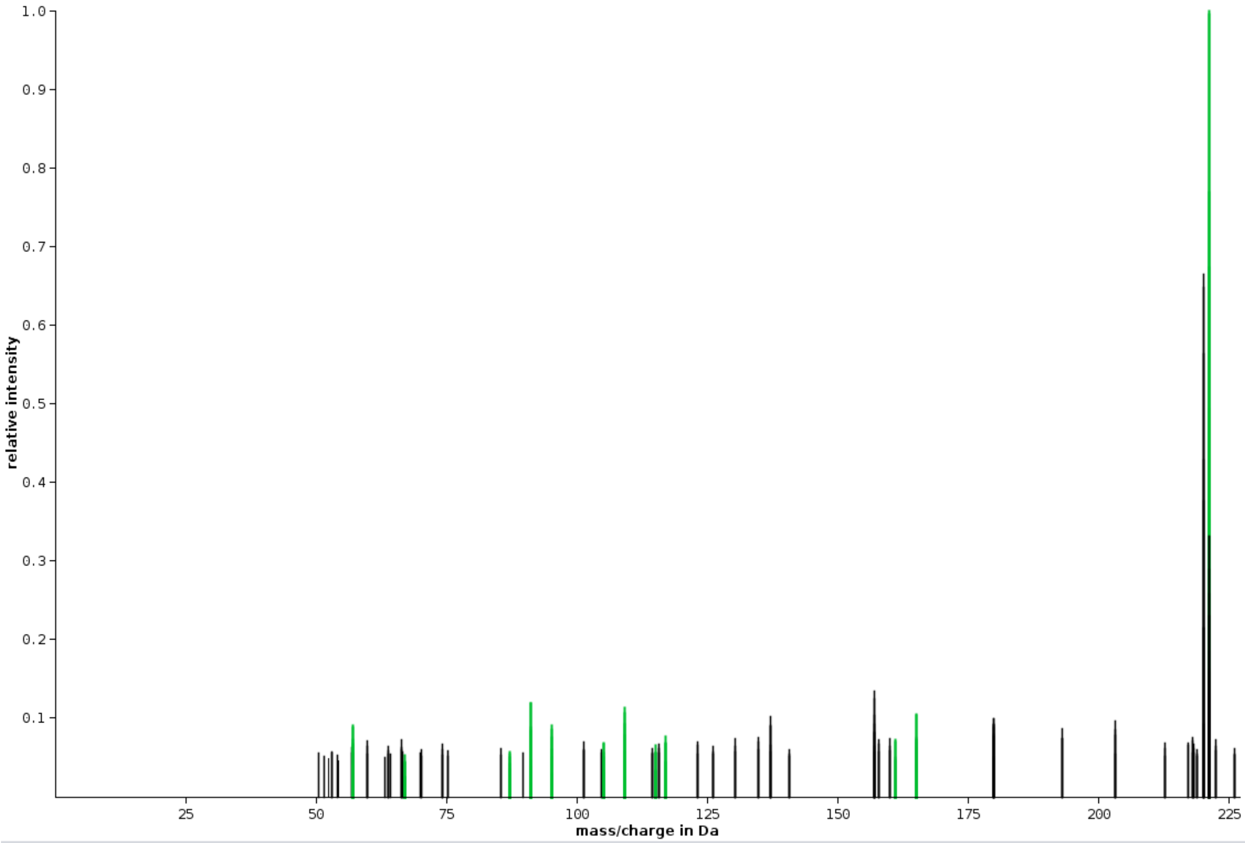

Figure S4. Fragmentation mass spectrum of the parent ion 221.0962 (m/z). Green lines indicate the peaks identified by SIRIUS.

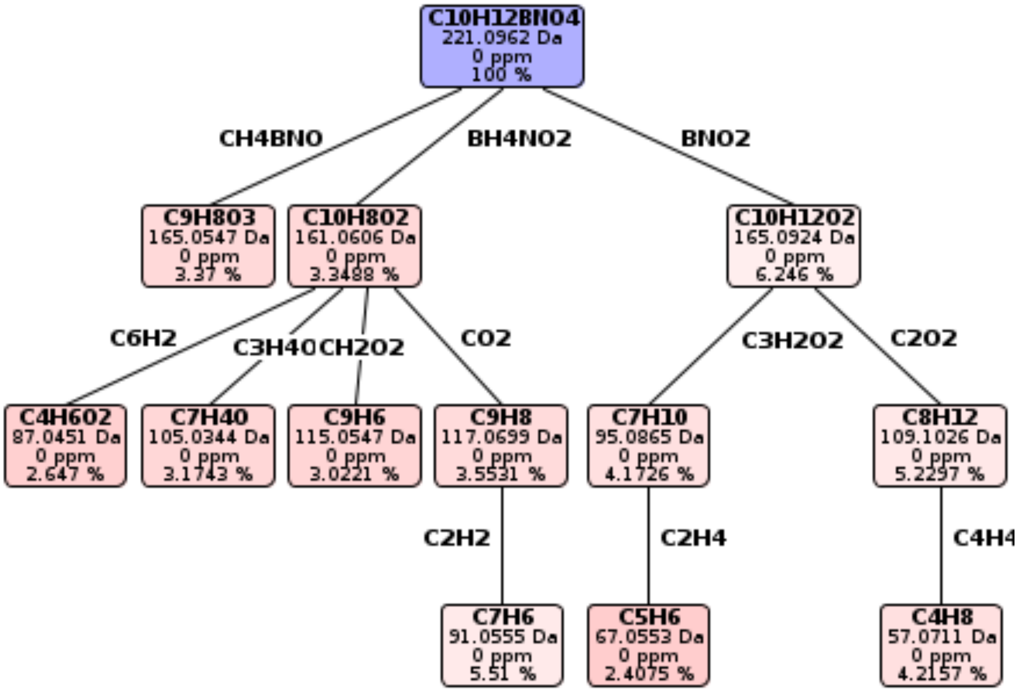

Figure S5. Fragmentation tree for the parent ion 221.0962 (m/z) generated by SIRIUS. Blue boxes indicate high intensity whereas red boxes indicate lower intensities. The molecular formula was predicted as $C_{10}H_{12}BNO_4$.

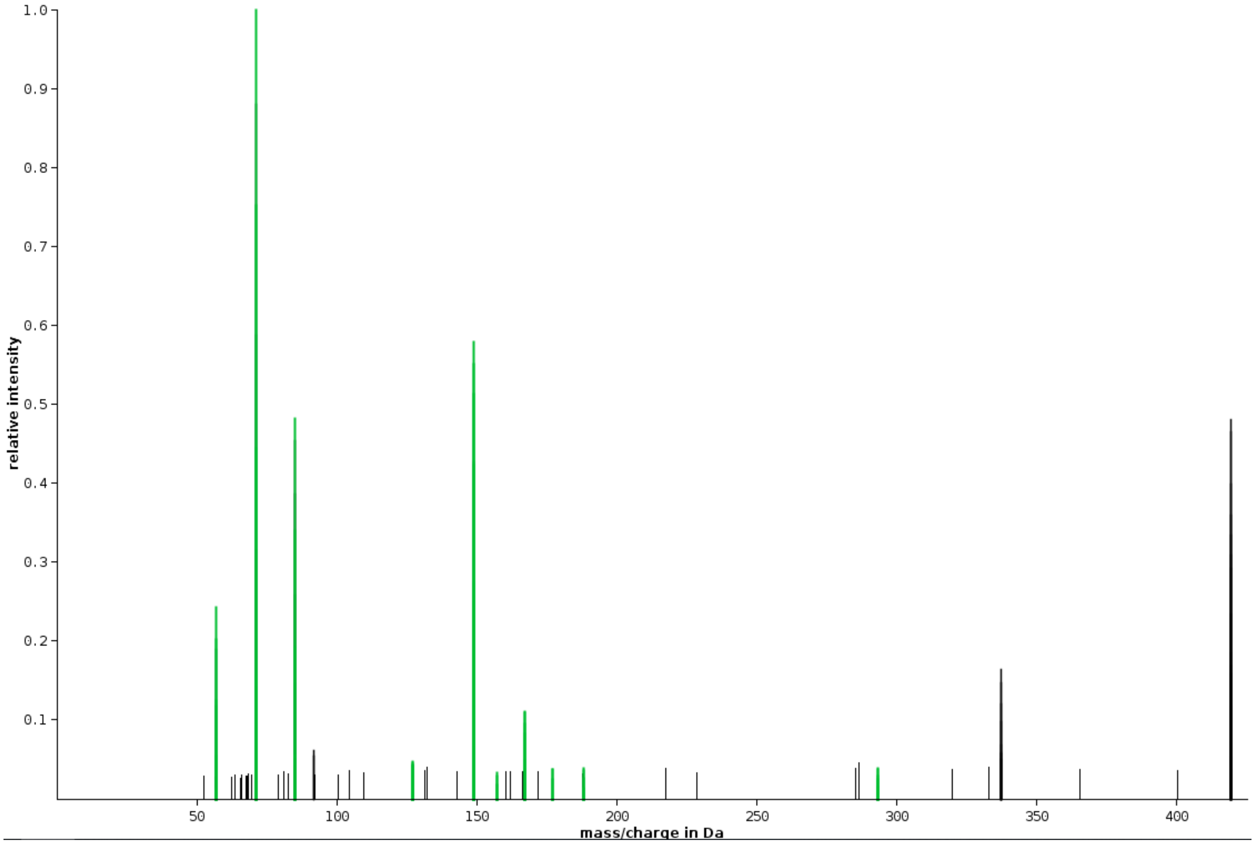

Figure S6. Fragmentation mass spectrum of the parent ion 419.3159 (m/z). Green lines indicate the peaks identified by SIRIUS.

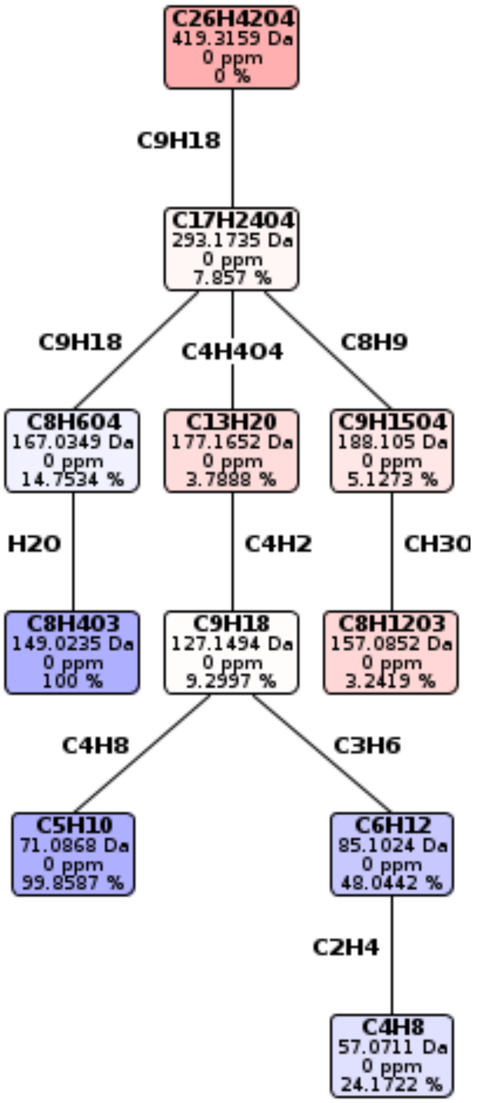

Figure S7. Fragmentation tree for the parent ion 419.3159 (m/z) generated by SIRIUS. Blue boxes indicate high intensity whereas red boxes indicate lower intensities. The molecular formula was predicted as $C_{26}H_{42}O_4$.

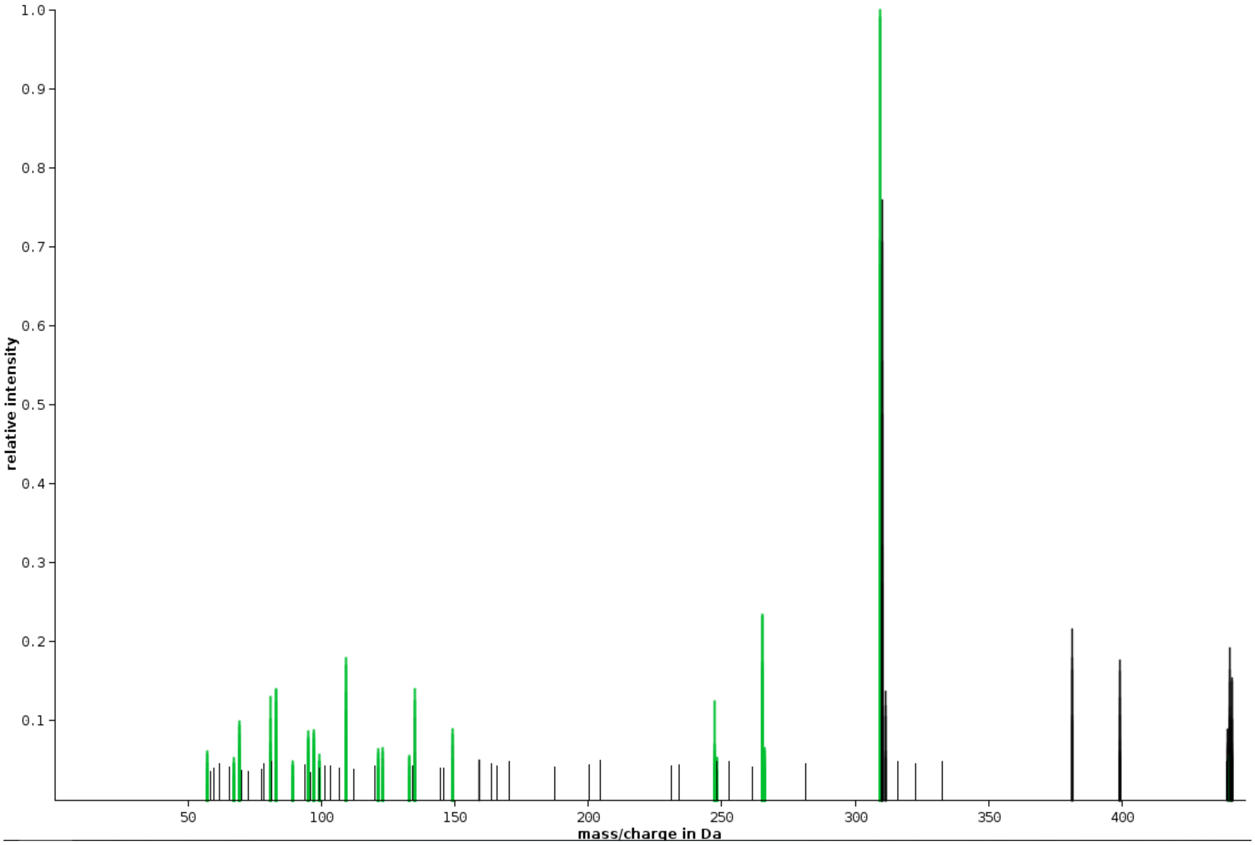

Figure S8. Fragmentation mass spectrum of the parent ion 440.3575 (m/z). Green lines indicate the peaks identified by SIRIUS.

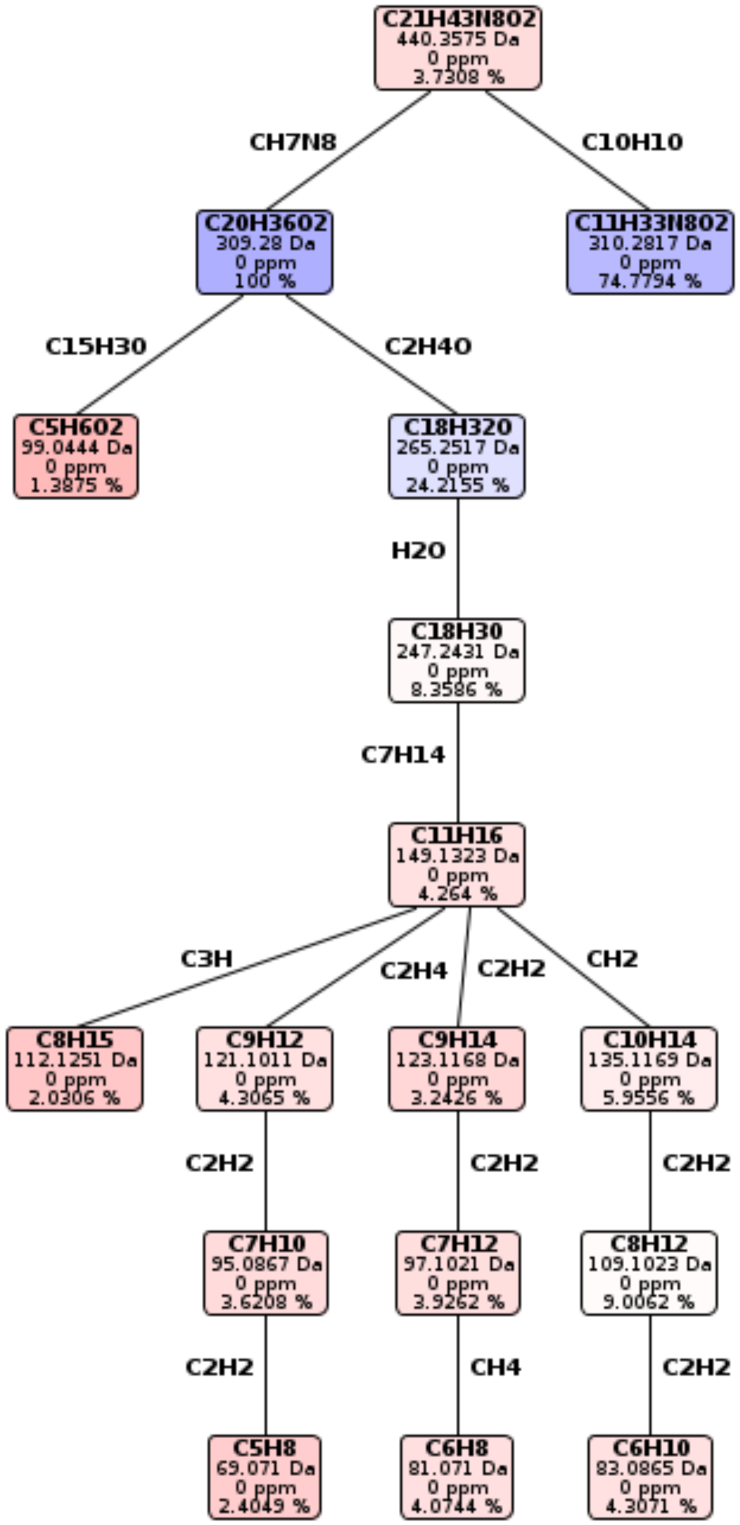

Figure S9. Fragmentation tree for the parent ion 440.3575 (m/z) generated by SIRIUS. Blue boxes indicate high intensity whereas red boxes indicate lower intensities. The molecular formula was predicted as $C_{21}H_{43}N_8O_2$.

References

1. Topçuoglu, B. D. *et al.* Hydrogen limitation and syntrophic growth among natural assemblages of thermophilic methanogens at deep-sea hydrothermal vents. *Front. Microbiol.* **7**, (2016).
2. ESRI. ArcGIS Desktop: Release 10.2. *Redlands CA* https://www.esri.com/en-us/arcgis/products/arcgis-enterprise/overview (2013).
3. Butterfield, D.A., V. Tunnicliffe, W.W. Chadwick Jr., J.A. Resing, J.E. Lupton, and M.D. Lilley, Geologic Setting and Fluid Chemistry May Drive Differences in Hydrothermal Vent Communities between the Mariana Arc and Back-Arc, in prep. for Frontiers in Marine Science.
